# Supplementary material for: Assessment of circulating microRNA specific for patients with familial adenomatous polyposis
Source: PLoS One. 2021 May 4;16(5):e0250072. doi: 10.1371/journal.pone.0250072 (PMC8096076; doi:10.1371/journal.pone.0250072)
Supplement: S1 Checklist — (PDF) [file pone.0250072.s001.pdf]

microRNA NGS Concluding Report

Project: PR0622 (Ref. code: 5223)

Performed by
Exiqon A/S
Company Reg.No.(CVR) 18 98 44 31
Skelstedet 16
DK-2950, Vedbæk
Denmark

Additional files provided:

File Name	Content
Data_ref_5223.xlsx	Summary of sequencing and mapping results<br>Contains counts used for the analysis (mapped to miRBase 20) for both microRNA and smallRNAs<br>Novel microRNAs based on prediction algorithm and not in standard miRBase or Rfam classification (miRPara).<br>Differential expression results from all comparisons of both microRNAs and predicted microRNAs<br>GO Analysis results<br>Custom analysis results (if performed)

Table 1. List of additional data files included with this report.

Files provided on disc drive:

An email containing information on encryption of disc drive will be sent, and the disc drive will be forwarded by courier.

Content	Description
Disk drive	High resolution copies of all pictures and graphs presented in this report. In either TIFF, PNG or PDF format<br>All FASTQ files associated with the project including FASTQC results<br>All BAM alignment files generated in the project including “mapped” and “unmapped” reads (use IGV viewer to visualize)<br>Read sets i.e. reads mapped to RFAM, reads “Out-mapped” mapped to empty vectors, mitochondrial, Poly C etc. and unmapped reads<br>Extensive results tables and Volcano plots for all differentials expression analysis<br>Extensive results tables and figures for all GO analysis<br>Unsupervised analysis for all group comparisons

Table 2. List of data files included on disc drive

Contents

Summary 4
Experimental overview 5
  Sample overview 5
  Reference genome 5
  Experimental design 5
Workflow 6
Data quality control 7
  QC Summary 7
  Adapter Trimming 9
  Spike-in QC 10
  Mapping 11
Results 13
  Number of reads 13
  Read types length distribution 14
  Identified microRNAs 15
  Normalization – TPM and TMM 16
  Principal Component Analysis plot 17
  Heat map and unsupervised clustering 18
  Identification of IsomiRs 19
  Differentially expressed microRNAs 20
  Comparison of experimental groups normal and FAP_patient 20
  Volcano plot 21
  NormFinder analysis 22
  Gene Ontology Enrichment Analysis 23
  Identification of novel microRNAs previously reported in other organisms 26
  Identification of novel microRNAs 27
Conclusion and next steps 28
Online search tool - miRSearch 29
Materials and methods 30
  Library preparation and Next Generation sequencing 30
Validating your NGS results 31
References 34
Frequently asked questions 35
Appendix 1 - Pipeline analysis overview 37

Summary

Dear Customer,

We have now finalized the next generation sequencing analysis of the microRNAs identified in the samples you have submitted. NGS sequencing libraries were successfully prepared, quantified and sequenced from all of your samples. The collected reads were subjected to quality control, alignment and downstream analysis. The principal findings are summarized in this document. Additional information and further details on specific microRNAs can be found in the various documents listed on the previous page.

Exiqon's miRCURY LNA™ product line offers many tools for further validating potentially regulated microRNAs by qPCR, in situ hybridization, or microRNA inhibition. For more information please follow the link: www.exiqon.com/mirna-products.

For further information on your microRNA of interest, please visit https://www.exiqon.com/mirsearch, our online search tool which quickly finds and displays detailed information about each microRNA.

If you have any questions related to this report, please do not hesitate to contact us at DxServices@exiqon.com.

Kind regards,

Exiqon Services

Exiqon A/S

Experimental overview

Sample overview

The table below lists all the samples processed in this project and their specifications according to the sample submission form. There were a total of 8 samples.

Customer ID	Groups	Fastq file name
PR0622_01_a_FAP-2-pre	FAP_patient;FAP-2-pre	5223-001-R1-c.fastq.gz
PR0622_02_a_FAP-6-pre	FAP_patient;FAP-6-pre	5223-002-R1-c.fastq.gz
PR0622_03_a_FAP-8-pre	FAP_patient;FAP-8-pre	5223-003-R1-c.fastq.gz
PR0622_04_a_FAP-10-pre	FAP_patient;FAP-10-pre	5223-004-R1-c.fastq.gz
PR0622_05_a_FAP-12-pre	FAP_patient;FAP-12-pre	5223-005-R1-c.fastq.gz
PR0622_06_a_FAP-N2	normal;normal_2	5223-006-R1-c.fastq.gz
PR0622_07_a_FAP-N3	normal;normal_2	5223-007-R1-c.fastq.gz
PR0622_08_a_FAP-N4	normal;normal_2	5223-008-R1-c.fastq.gz

Table 3. Sample ID, grouping, and associated file.

Reference genome

Annotation of the obtained sequences was performed using the reference annotation listed below.

Organism: *Homo sapiens*

Annotation reference: miRbase 20 (http://www.mirbase.org/)

Experimental design

The experiments were performed using the following settings:

Instrument: Nextseq500

Average number of reads: 10 mio. reads / per sample

Number of sequencing cycles (read length): 50 nt. Single-end read

Workflow

The figure below describes the Next Generation Sequencing process for biofluids microRNA and small RNA sequencing at Exiqon.

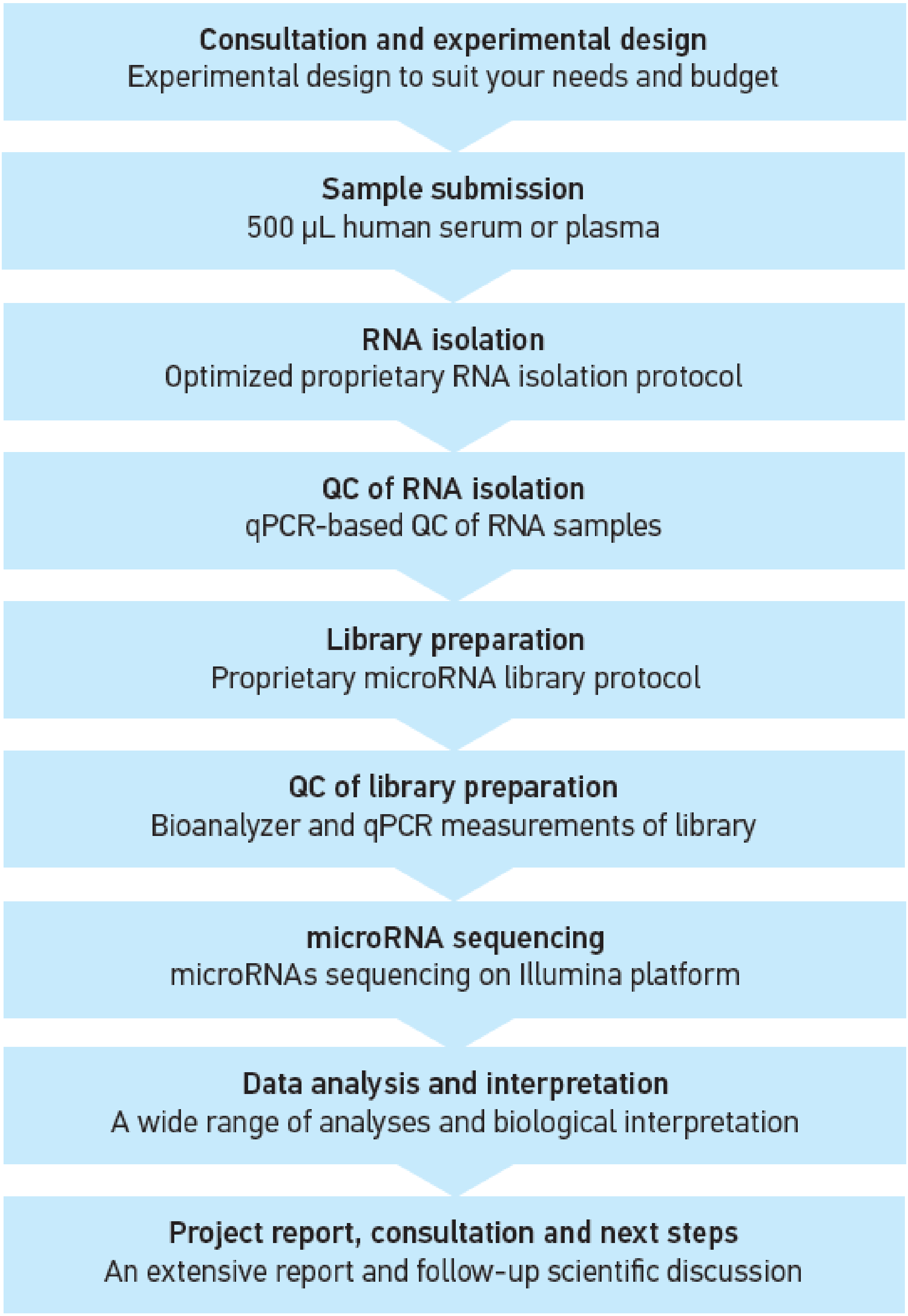

Figure 1. Schematic of NGS workflow.

Data quality control

QC Summary

Following sequencing, intensity correction and base calling and assigning of Q-scores is performed. Subsequenctly the data is quality checked The samples showed overall good data quality with the vast majority of the data obtained, presenting higher Q-score than Q30.

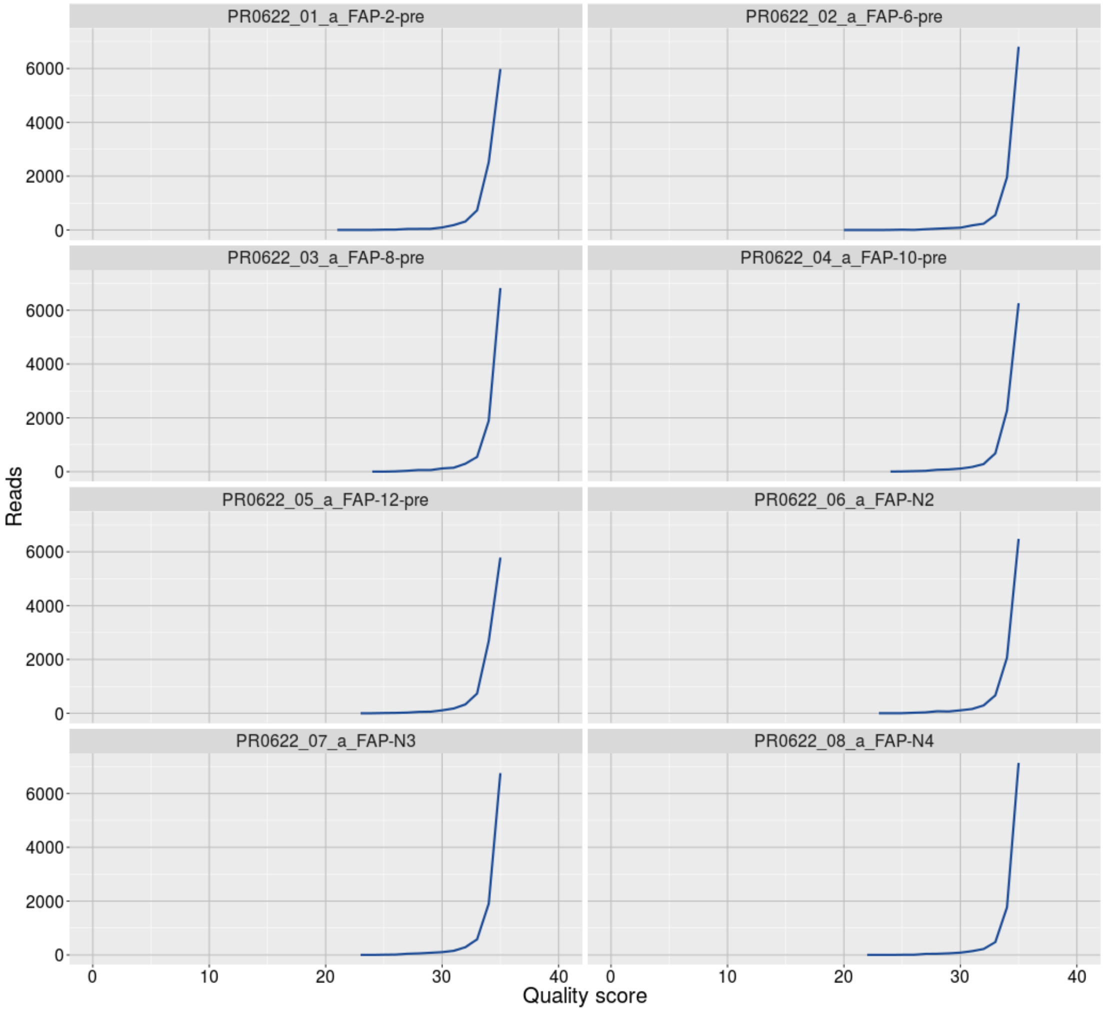

Figure 2. The average reads Q-scores of the NGS sequencing data for the samples. The vast majority of the data has Q score greater than 30.

Q-score
The Q-score (or quality score) is a prediction of the probability of an incorrect base-call. A Q-score of 30 equals an accuracy of 99.9% for the base-calling (Cock et al., 2010).

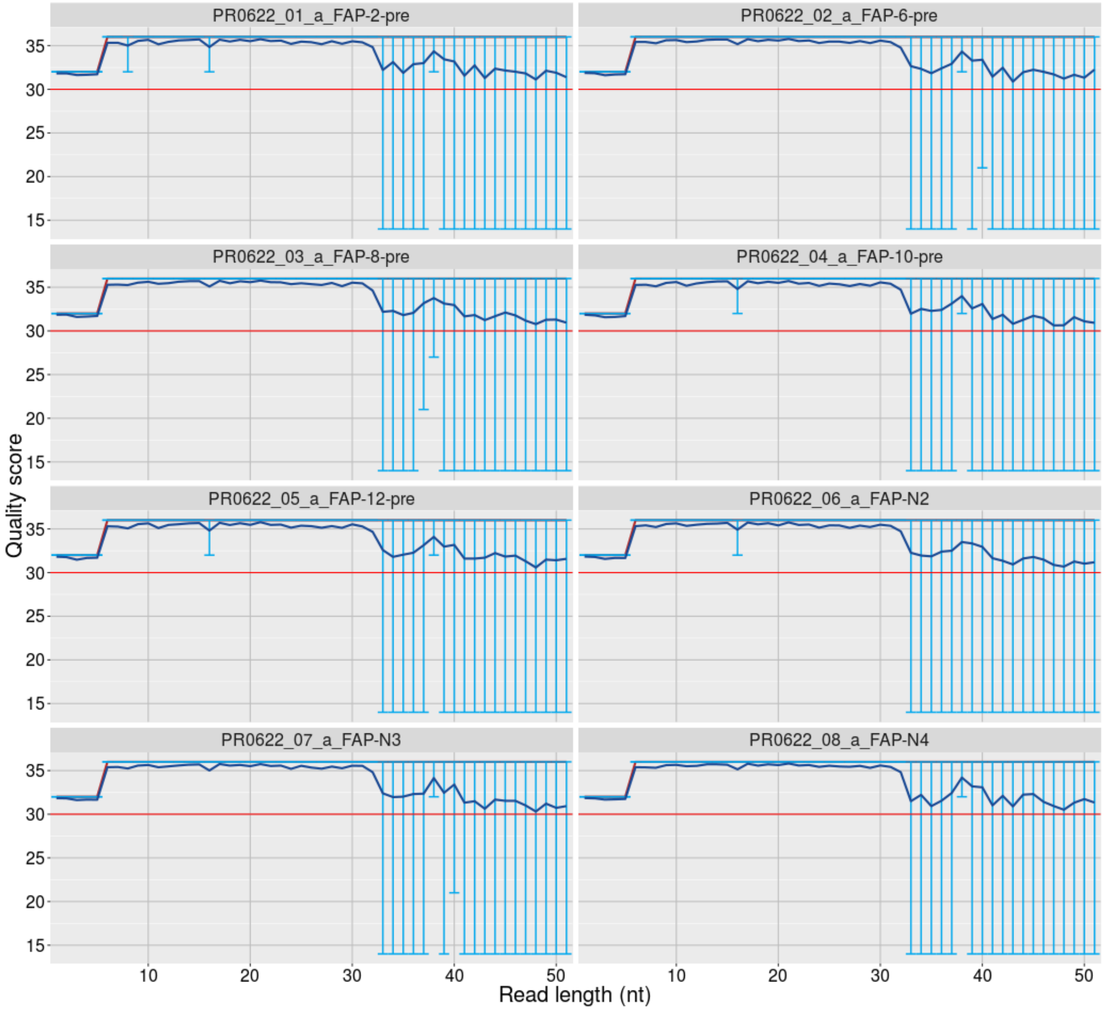

Figure 3. The average base Q-scores of the NGS sequencing data for the samples. The vast majority of the data has a Q score greater than 30 (>99.9% correct). Please note that the sequencing instrument bins the quality score in order to save space, the quality score is binned in to 7 categories based on their Q-score. For further information refer to the Illumina technote - Understanding Illumina Quality Scores (http://www.illumina.com/documents/products/technotes/technote_understanding_quality_scores.pdf)

Adapter Trimming

After sequencing adapters were trimmed off as part of the base calling. Trimming of adapters from the dataset revealed distinct peaks representing microRNA (~18-23nt), and longer sequences of other origin (i.e. YRNA, rRNA, tRNA and mRNA fragments, ~30-50nt).

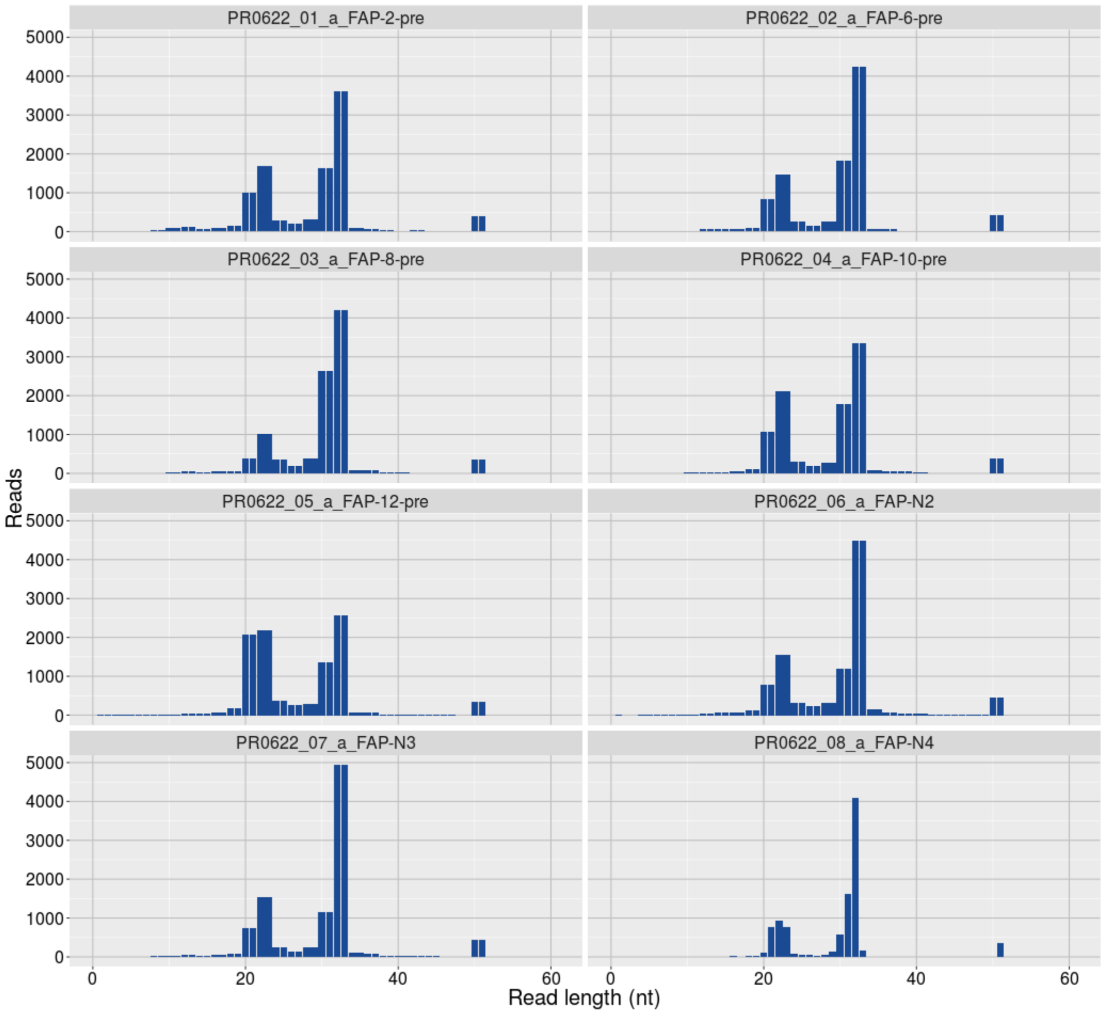

Figure 4. Read length distribution after filtering of the adaptors: The samples have the expected peak around 18-23 nt. representing microRNAs and longer sequences of other origin (i.e. YRNA, rRNA, tRNA and mRNA fragments, ~30nt).

Spike-in QC

A range of spike-ins was added to the samples prior to RNA isolation. We observe an excellent correlation of counts corresponding to the spike-ins between the samples (>0.98 on average). The actual R^2 values are reported in the spikein_correlation_report.pdf in the supplementary files. A visualization of the spike-in reads in the samples can be seen in the figure below.

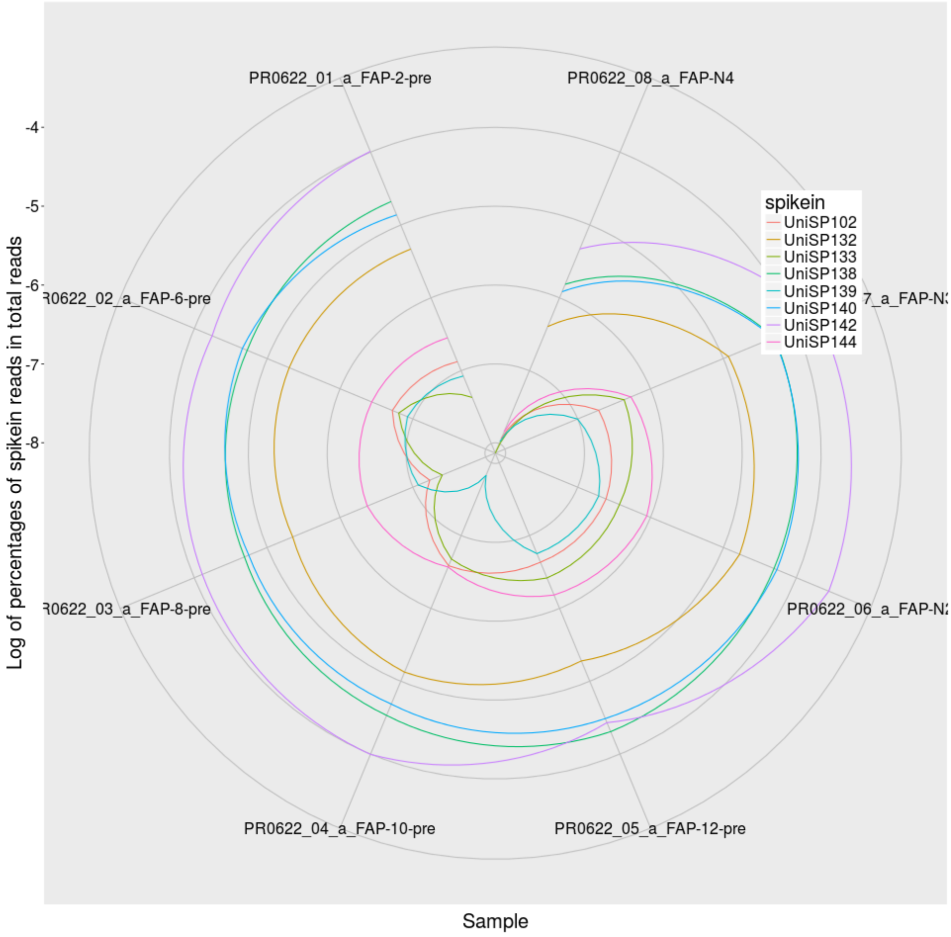

Figure 5. Radar plot showing relative spike-in signal for the samples.

Mapping

Mapping of the sequencing reads is the first part of the data analysis but it also represents a useful quality control step in the NGS data analysis pipeline as it can help to evaluate the quality of the samples. For this purpose, we classify the reads in the following classes:

**Outmapped**:	Reads that aligned to abundant sequence, for example polyA and PolyC homopolymers. This also includes reads that align to ribosomal RNA, the mitochondrial chromosome, and the genome of phiX174. These reads are discarded and are not used in the analysis.
**Unmapped reads**	Reads that could not be alignment to reference genome. There are several possible explanations as to why some reads fail to align to the reference genome.
**Genome**:	Reads that align to the reference genome in locations where *no* known microRNAs or smallRNAs are located. This also included fragments of mRNA and lncRNA transcripts
**MicroRNA**:	Reads mapped to miRBase.
**SmallRNA**	Reads mapped to smallRNA database.
**Predicted (pred)**:	There are two different kinds of predicted microRNA; Either the sequence is found in miRBase in another organism (prediction based on sequence homology) or the sequence is predicted to be microRNA by miRPara prediction algorithm.

A typical biolfluids microRNA sequencing experiment yields approximately 70-90% of the reads mapping to the reference genome. 10-60% of the mappable reads are typically annotated to microRNAs. However, this is dependent on the quality of the sample and how well of the reference genome is characterized, as well as the microRNA annotation in miRBase. If the sample is degraded fewer reads will be microRNA specific and more material will be degraded rRNA/tRNA. The following plot summarizes the overall mapping results.

The plot shows that the fraction of mappable reads (=counts) is within the normal range, and that the distribution is relatively similar for all samples.

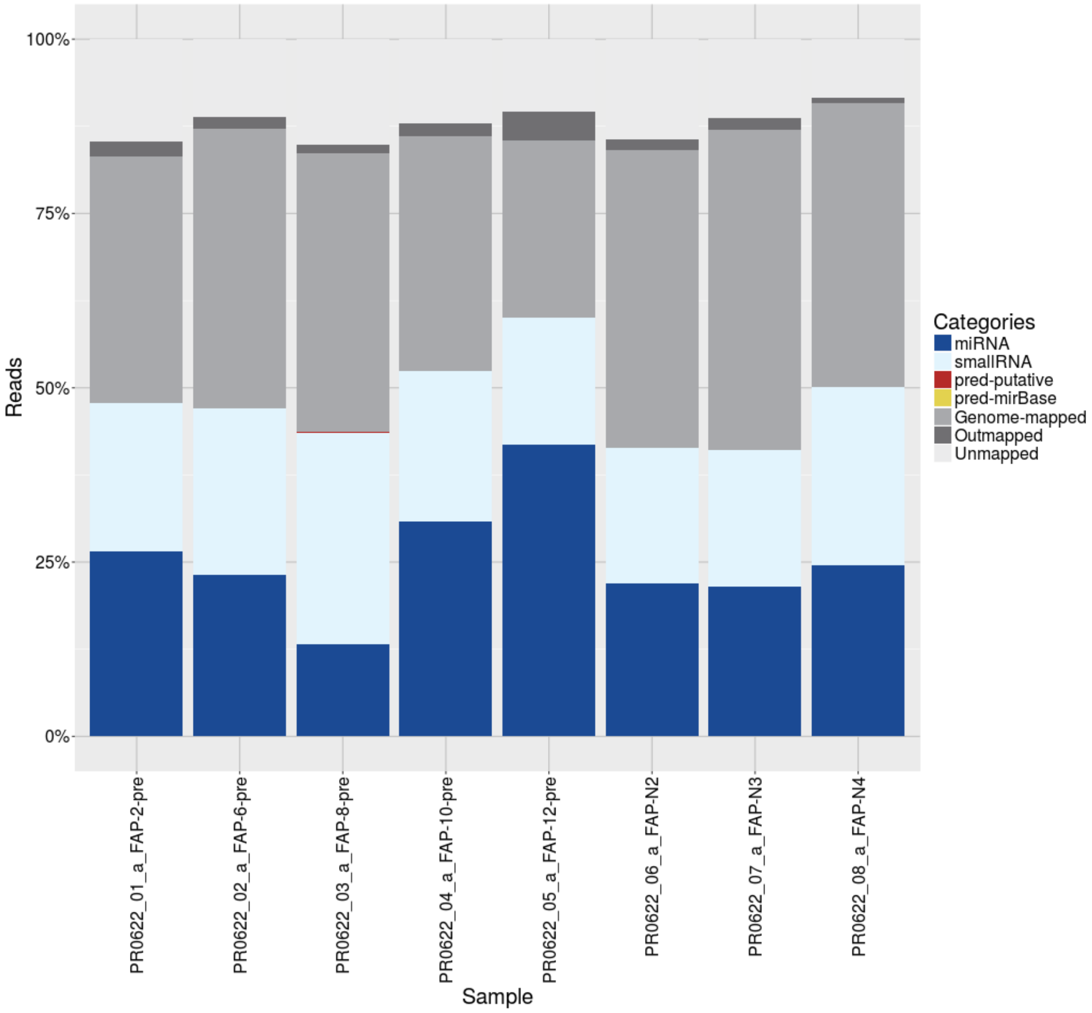

Figure 6. Summary of mapping of NGS counts for each sample in the project.

Inspection of mapped data
If you want to inspect the mapping in details, you can use the BAM alignment files together with the BAM index file, which are supplied on the hard disk. The BAM files can be viewed and inspected in any standard genome viewer such as the IGV browser (Robinson et al. (2011) and Thorvaldsdóttir et al. (2012)). IGV can be downloadable from:
http://software.broadinstitute.org/software/igv/
Please note that NGS data requires powerful computer resources and might cause I a standard desktop or laptop to freeze. A full user guide of the IGV browser can be found here:
http://software.broadinstitute.org/software/igv/UserGuide
Especially the part about Downsampling in the Viewing Alignment part can be useful for looking at the data.

Results

The following sections provide a summary of the data analysis performed on your dataset. The complete analysis results may be found in the associated files listed on page 2.

Number of reads

The NGS profiling was successfully completed for your samples. On average 11.7 million reads were obtained per sample. The figure below shows the total number of reads per sample.

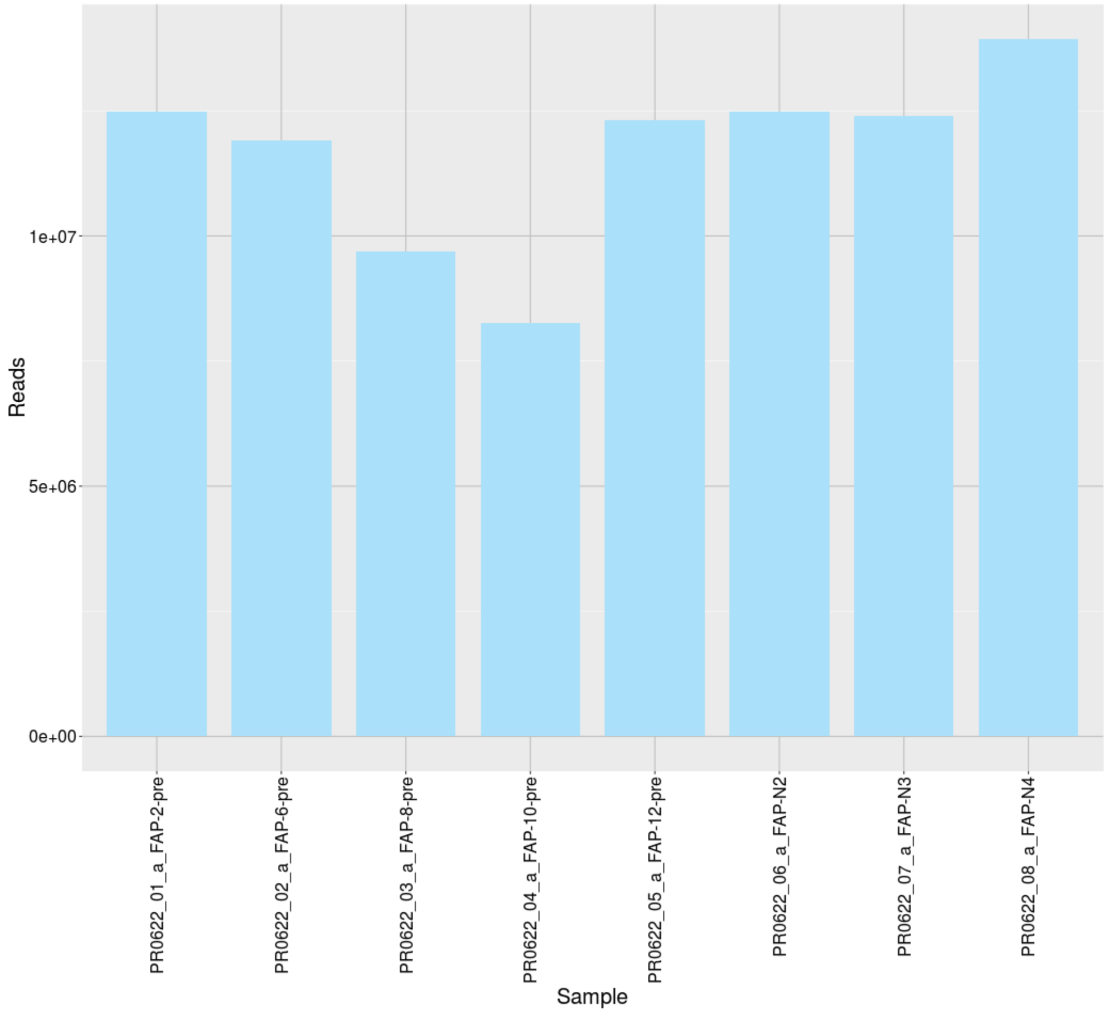

Figure 7. The total number of reads for each sample sequenced in this project. On average 11.7 million reads were obtained from each sample.

Read types length distribution

After mapping of the data each read is assigned to a class of RNA and the length distribution of the reads is presented in the figure below with RNA from each class separately.

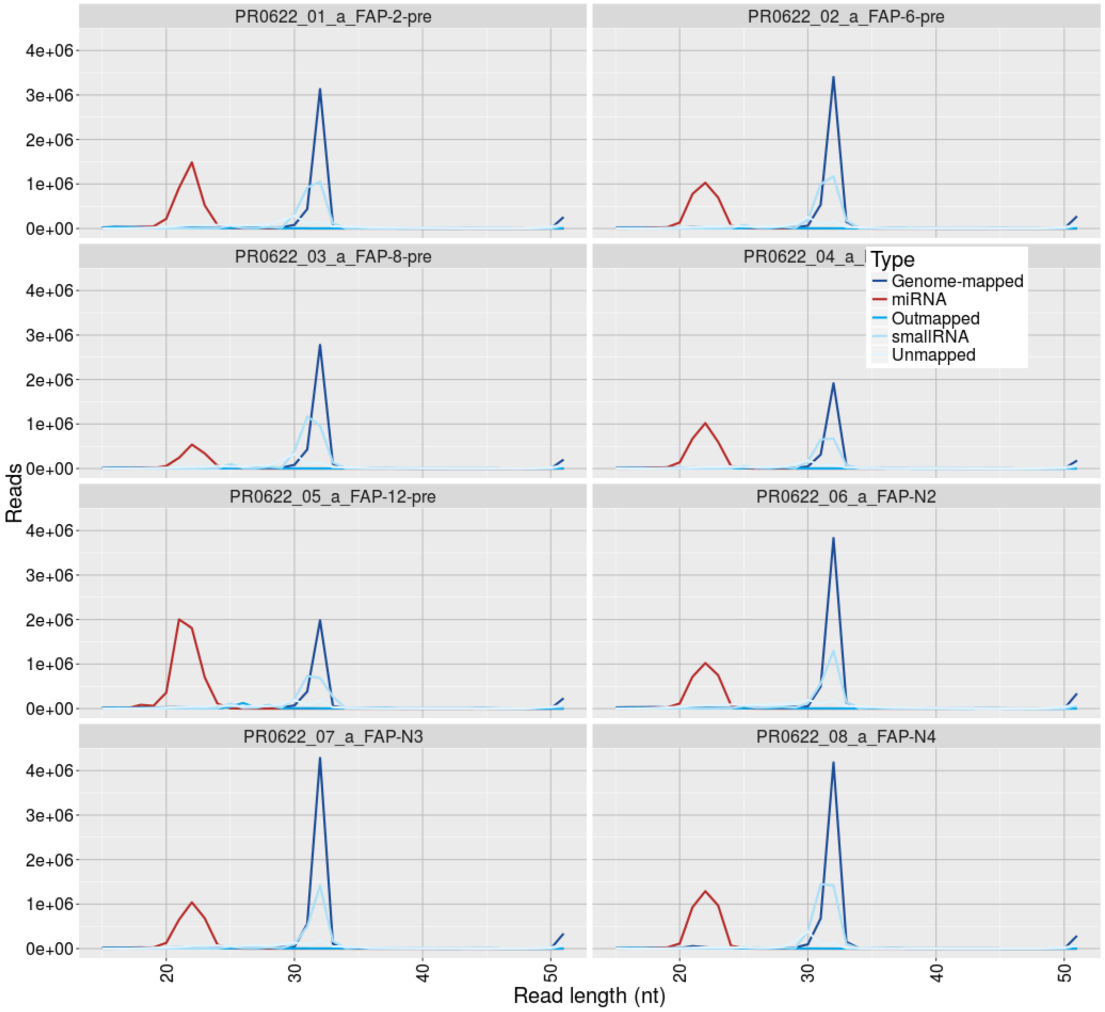

Figure 8. Read length distribution for each class of RNAs: The samples have the expected peak for microRNA around 18-23 nt. A peak around 30 nt. is also seen which is common in biofluid NGS experiments containing degraded RNA molecules (mostly YRNA and tRNA fragments).

Identified microRNAs

After mapping the data and counting to relevant entries in miRBase 20 the numbers of known microRNAs was calculated. The reliability of the identified microRNAs increased with number of identified fragments. When performing the statistical comparison of two groups, we include all microRNAs irrespective of how few calls have been made

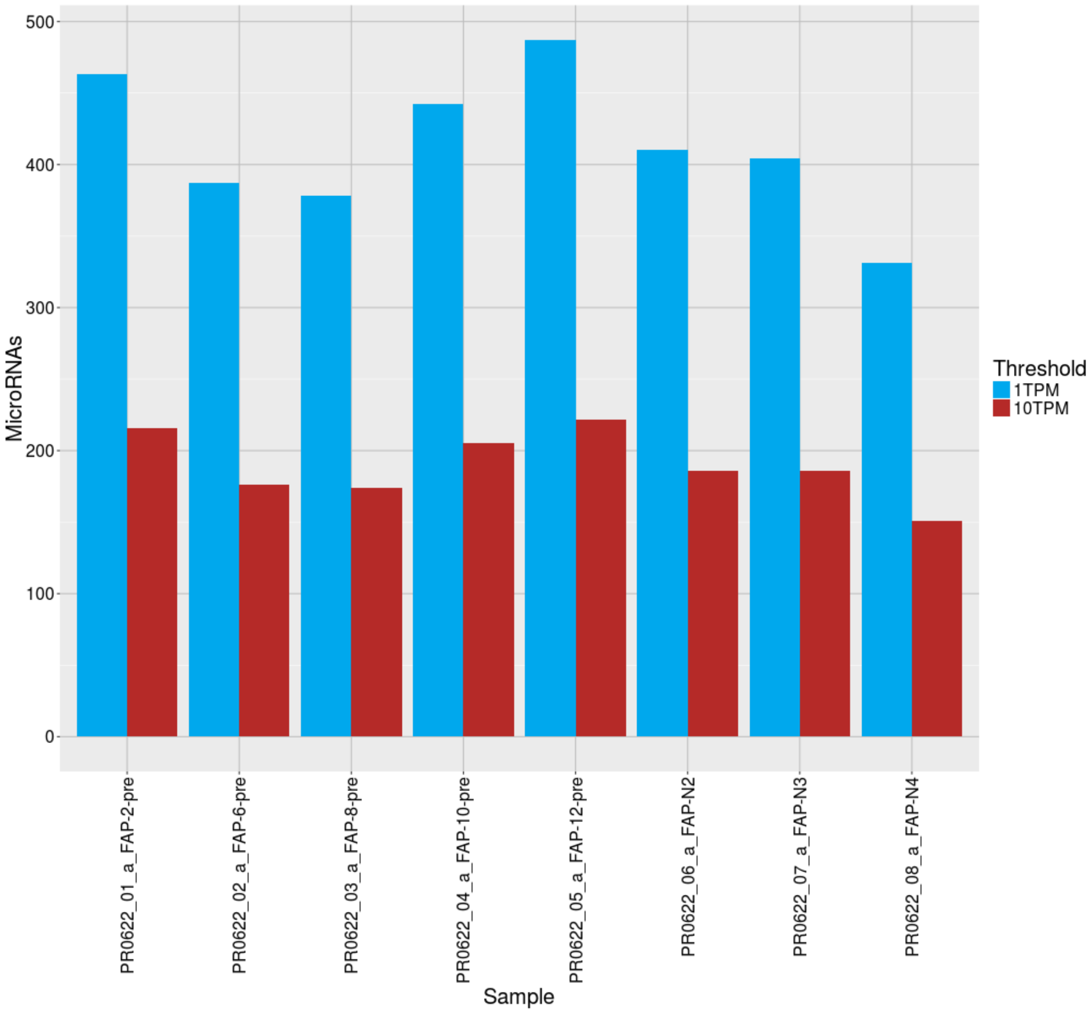

Figure 9. Number of identified known microRNAs with number of counts (>1TPM per sample in blue bars and >10TPM per sample in red bars).

Grouping	Quantity (all samples)
**Number of identified RNAs in all samples (≥ 1 TPM)**	382
**Number of identified RNAs in all samples (≥ 10 TPM)**	198

Table 4. Summary of identified microRNAs across all samples accepted for the analysis. microRNAs are identified according to entries in miRBase release 20.

Expression levels are measured as Tags Per Million ("TPM")
TPM is a unit used to measure expression in NGS experiments. The number of reads for a particular microRNA is divided by the total number of mapped reads and multiplied by 1 million (Tags Per Million).

Normalization – TPM and TMM

TPM is a unit used to measure expression levels in NGS experiments and can be used for any non-fragmenting library protocol. The number of reads that map to a particular RNA species is divided by the total number of mapped reads in the sample and subsequently multiplied by one million. This is a simple normalization procedure that corrects for the sequencing depth and provides a very transparent measure of quantity for each RNA species.

However, for the statistical analyses presented in this report the trimmed mean of M-values normalization method (TMM normalization) is used (Robinson and Oshlack, 2010). Like TPM normalization, TMM normalization compensate for sample specific effects caused by the variation in library size/sequencing depth between samples. In contrast to TPM, the TMM normalization step also compensate potential under- and over-sampling effects by trimming and applying scaling factors that minimize log fold changes between samples across the majority of the microRNAs.

If only applying TPM normalization, differential gene expression of the samples' microRNA population will result in libraries with biases e.g. when a few highly expressed microRNAs dominate the read set in one sample type and not in the other. (In an experiment with a fixed number of sequenced reads, e.g. 10 million reads per sample, fewer reads will "remain" to cover RNA species with constant expression levels if one or a few specific RNA species increase significantly from one biological condition to another. If uncorrected, such effects could lead to skewed analyses and apparent downregulation of RNA species which are, in fact, constantly expressed. TMM normalization attempts to reduce the sample-to-sample effects caused by significant differences in expression levels of a subset of RNA species).

The differential expression analysis is done using TMM in the EdgeR statistical software package (Bioconductor, http://www.bioconductor.org/). Differential expression analysis on TMMs investigates the relative change in expression (i.e. counts) between different samples and with TMM normalization the statistical tests will be less skewed and the false-positive rate is reduced. Note that for each differential expression comparison, TMM is calculated, based on the subset of features and subset of samples analyzed. The Average TMM values per group and individual values per Sample are presented in the DE_XXX sheets in the supplied excel document.

It is difficult to calculate back from a TMM value into a number of aligned reads. However, the benefits to the downstream analyses outweigh the drawbacks caused by loss of transparency. As standard, Exiqon Services also perform TPM (tags per million) normalization as this normalization procedure is easier to interpret to an actual expression level for a microRNA in a given sample. The TPM normalized data is provided in the data tables accompanying this report.

Principal Component Analysis plot

Principal Component Analysis (PCA) is a method used to reduce the dimension of large data sets and thereby a useful way to explore the naturally arising sample classes based on the expression profile. Here, by including the top 50 microRNAs that have the largest variation across all samples, an overview of how the samples cluster based on this variance is obtained. The data is normalized with the trimmed mean of M-values (TMM) method and converted to a log2 scale. Then all features are filtered on “expressed in all samples” criteria and the 50 features with the highest coefficient of variation (%CV) selected for the analysis. If the biological differences between the samples are pronounced, this will be a primary component of the variation. This leads to separation of samples in different regions of a PCA plot corresponding to their biology. If other factors, e.g. sample quality, inflict more variation on the samples, the samples will not cluster according to the biology. The largest component in the variation is plotted along the X-axis (this is often referred to as the first principle component or “PC1” for short) and the second largest component in the variation is plotted on the Y-axis (this is often referred to as the second principle component or “PC2” for short).

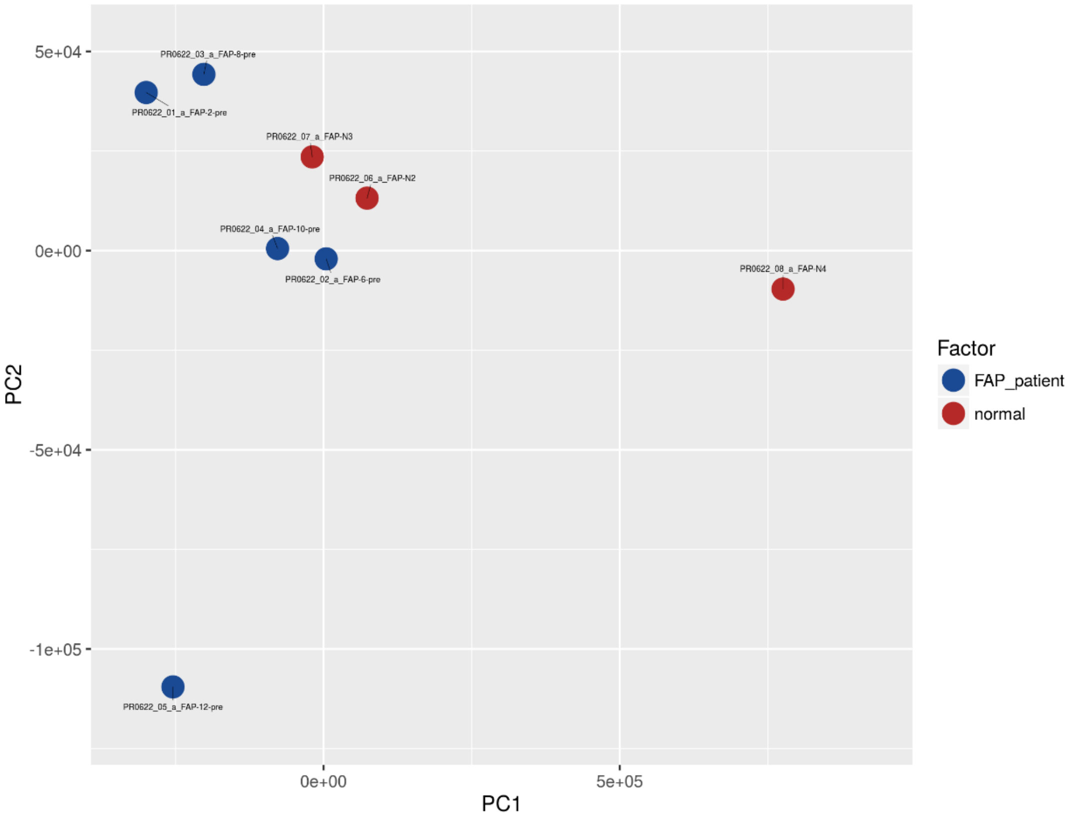

Figure 10. Principal component analysis (PCA) plot. The PCA was performed on all samples passing QC using the top 50 microRNAs with highest CV (based on TMM normalized reads).

Heat map and unsupervised clustering

The heat map diagram in the figure below shows the result of the two-way hierarchical clustering of microRNAs and samples. The data is normalized with the trimmed mean of M-values method and converted to a log2 scale. Then all features are filtered on "expressed in all samples" criteria and the 50 microRNAs with the highest coefficient of variation (%CV) selected for the analysis. If the biological differences between the samples are pronounced, this will be a primary component of the variation. This leads to separation of samples in different clades in the heatmap corresponding to their biology. If other factors, e.g. sample quality, inflict more variation on the samples, the samples will not cluster according to the biology. Each row represents one microRNA, and each column represents one sample. The color of each point represents the relative expression level of a microRNA across all samples: The color scale shown at the bottom right: Red represents an expression level above the mean, green represents an expression level below the mean.

Other short RNA species than microRNAs are excluded from this plot even if they show high % CV across all samples.

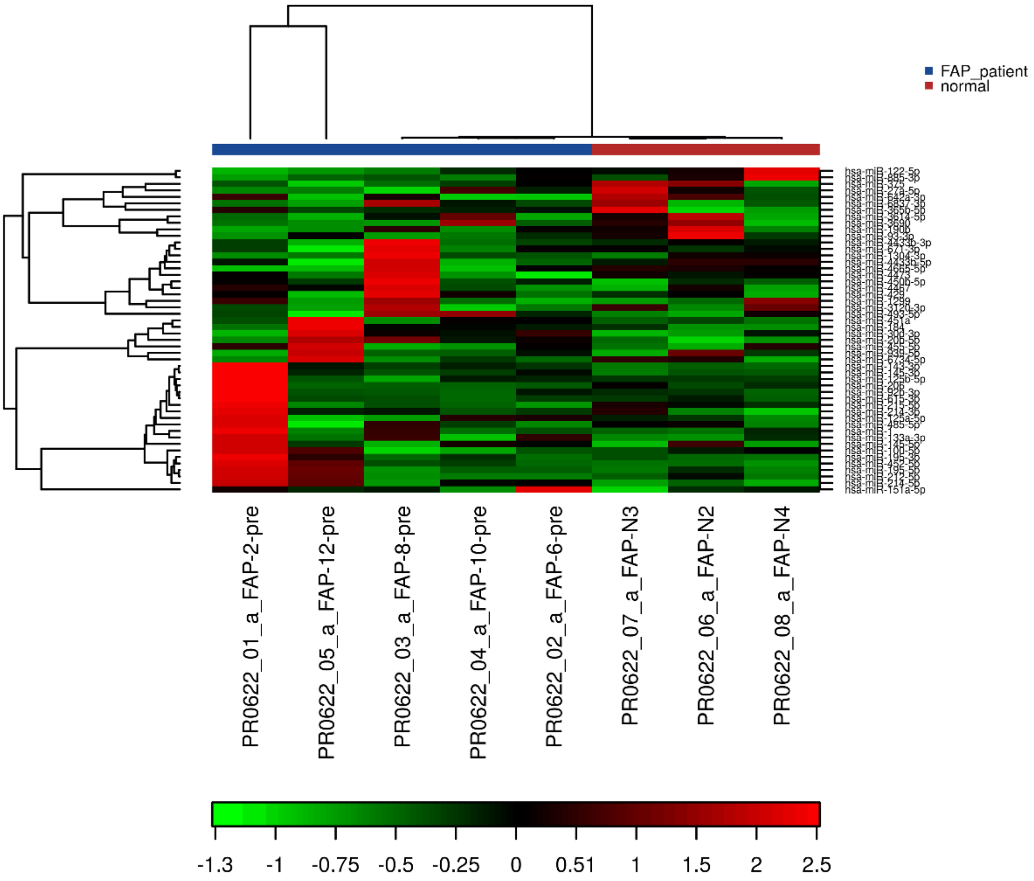

Figure 11. Heat Map and unsupervised hierarchical clustering by sample and microRNA. The clustering was performed on all samples, and on the top 50 microRNAs with highest CV based on TMM normalized counts.

Identification of IsomiRs

IsomiR analysis is performed individually for each sample based on the occurrence of count variants for each detected microRNA. Reads are mapped to known microRNAs according to the annotation in miRBase release 20 and then investigated for the presence of different isomiRs. These variants are identified by changes in start or stop position, or occurrence of mutations within the read. The results for each sample are then merged to generate a single count file with a consistent nomenclature across the samples. Only isomiRs that are present at a level of 5% of total reads for that miRNA are retained. No differential expression analysis is performed on the isomiR's, but the count file can give an impression of differences in abundance.

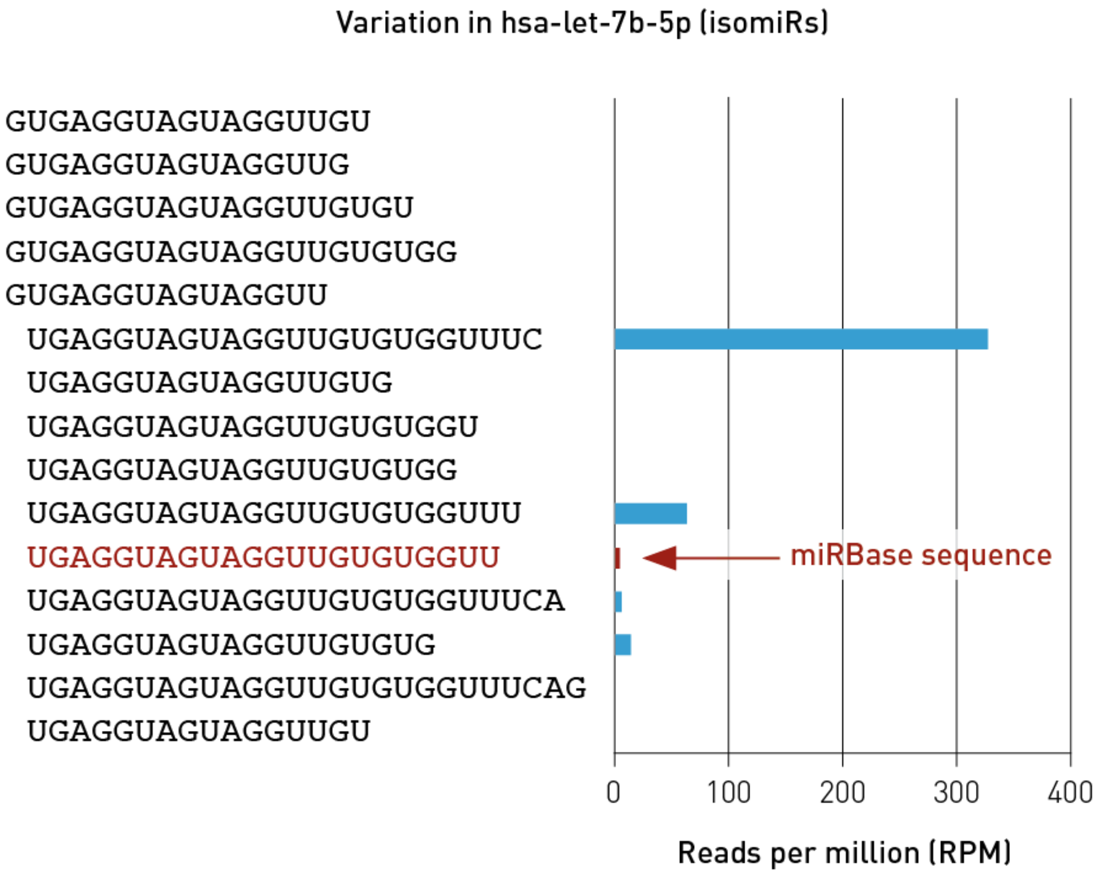

Figure 12. Example of differences in isomiRs in a NGS experiment. isomiR data for this project can be found in the supplementary files in the mircount_data folder.

Because of the large amount of information generated in an isomiR analysis, the results are split into several sheets in the 'mircount_data' folder and it's subfolders to provide both detailed and summary information for the data.

Differentially expressed microRNAs

The differential expression analysis step attempts to distinguish biological variation from technical variation within the experiment, assuming that this varies amongst microRNAs. p-values for significantly differentially expressed microRNAs are estimated by an exact test on the negative binomial distribution. A list of microRNAs predicted to be differentially expressed between the given experimental conditions is presented below.

Comparison of experimental groups normal and FAP_patient

The table below shows the individual results for the twenty most differentially expressed microRNAs. A full list of differentially expressed microRNAs is given in the associated Data_Ref_5223.xlsx spreadsheet.

names	logFC	logCPM	PValue	FDR	normal	FAP_patient
hsa-miR-143-5p	-2.764	3.060	0.000445	0.085001	2	11
hsa-miR-96-5p	-3.295	2.998	0.00048	0.085001	1	11
hsa-miR-218-5p	-2.935	4.618	0.001686	0.13342	5	35
hsa-miR-106a-5p	-2.196	3.137	0.00177	0.13342	2	11
hsa-miR-10b-3p	-2.205	3.894	0.002404	0.13342	5	20
hsa-miR-143-3p	-2.492	13.999	0.002771	0.13342	4208	23673
hsa-miR-885-5p	2.828	2.909	0.002913	0.13342	15	2
hsa-miR-183-5p	-1.690	10.043	0.003298	0.13342	441	1422
hsa-miR-214-5p	-2.430	4.402	0.003392	0.13342	6	29
hsa-miR-885-3p	1.777	3.818	0.008731	0.306145	25	7
hsa-miR-122-5p	1.892	18.297	0.009513	0.306145	592567	159690
hsa-miR-20b-5p	-1.600	5.034	0.013132	0.37398	14	43
hsa-miR-93-3p	1.574	3.221	0.013734	0.37398	15	5
hsa-miR-145-3p	-1.878	8.027	0.019149	0.46053	97	357
hsa-miR-342-3p	1.167	5.666	0.019514	0.46053	77	34
hsa-miR-184	-1.885	3.531	0.02181	0.463537	4	15
hsa-miR-146a-3p	2.081	3.039	0.02226	0.463537	15	4
hsa-miR-1180-3p	-1.183	7.052	0.023826	0.46858	74	167
hsa-miR-452-5p	-1.709	3.138	0.027128	0.489492	4	11
hsa-miR-16-2-3p	-1.179	10.326	0.027655	0.489492	716	1623

Table 5: Table of the 20 most significantly differentially expressed microRNA and annotation, with log fold change (logFC) between groups; normal and FAP_patient, raw p-values, Benjamini-Hochberg FDR corrected p-values as well as the average TMM values per group. Please note that comparisons can yield highly significant hits even though the comparison is done on the basis of very few observed reads so pay attention to the counts as well as the p-values and logFC. The full table is presented in the Data_Ref_5223.xlsx spreadsheet file.

Additional comparisons performed for this project
Only one comparison is presented in this summary report. For a full list of comparisons see the file Data_Ref_5223.xlsx. The file contains summary of all the comparisons in separate sheets (tabs). The full results from all comparisons can be found on the accompanying hard disk.

Volcano plot

The Volcano plot provides a way to perform a quick visual identification of microRNAs displaying large-magnitude changes which are also statistically significant. The plot is constructed by plotting the p-value (-log10) on the y-axis, and the expression fold change between the two experimental groups on the x-axis. There are two regions of interest in the plot: those points that are found towards the top of the plot (high statistical significance) and at the extreme left or right (strongly down and up-regulated respectively). A full list of differentially expressed micro/smallRNAs is given in the associated Data_ref_5223.xlsx spreadsheet.

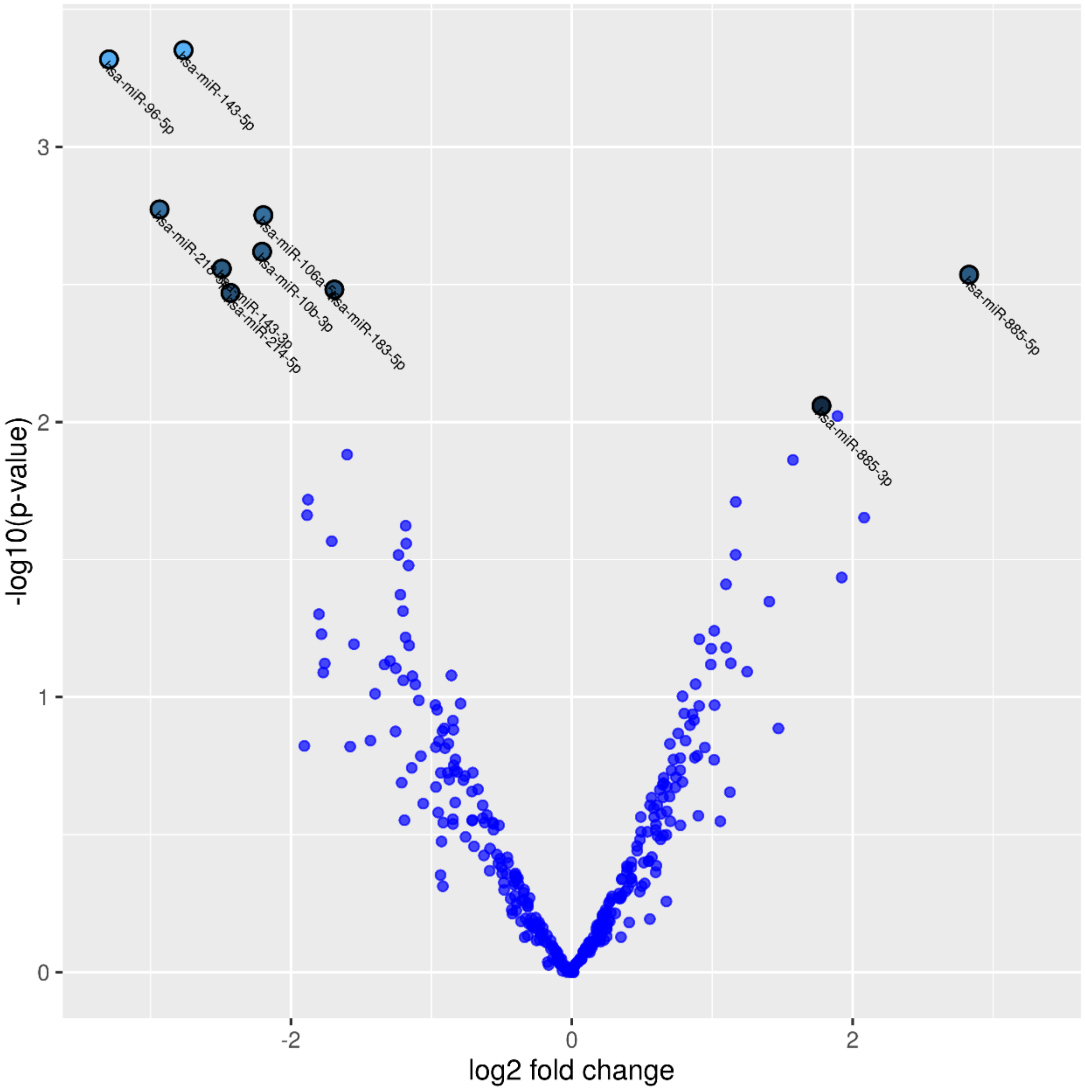

Figure 13. Volcano plot showing the relationship between the p-values and the fold change in normalized expression between the experimental groups; normal and FAP_patient. microRNAs with p-values below 0.05 are marked with names on the plot

NormFinder analysis

If microRNAs were found differentially expressed in the NGS analysis these findings should be validated, e.g. by a qPCR study. The table below shows which microRNAs were most stably expressed across the two groups and may thus be good candidates for normalizers/house-keeping genes in qPCR validation study.

Name	Stability	TPM average
hsa-miR-16-5p	26.51	215
hsa-miR-30e-5p	26.51	457
hsa-miR-140-3p	26.67	453
hsa-miR-186-5p	26.67	415
hsa-miR-106b-3p	27.21	318
hsa-miR-26b-5p	27.95	403
hsa-miR-378a-3p	28.72	423
hsa-miR-29a-3p	29.11	202
hsa-miR-182-5p	31.48	306
hsa-miR-16-2-3p	33.74	434
hsa-miR-183-5p	33.74	370
hsa-miR-363-3p	36.47	322
hsa-miR-181a-5p	41.65	424
hsa-miR-629-5p	49.2	112
hsa-miR-22-3p	61.15	739
hsa-miR-361-3p	71.81	524
hsa-miR-27a-3p	71.84	685
hsa-miR-30a-5p	71.84	517
hsa-miR-185-5p	73.41	185
hsa-let-7c-5p	74.38	540
hsa-miR-532-5p	82.93	794
hsa-miR-486-3p	88.84	177
hsa-miR-342-5p	101.12	165
hsa-miR-191-5p	125.48	985
hsa-miR-148b-3p	138.69	398

Table 6. Table of the 25 most stably expressed miRs across he sample set. Stability is the stability measure. Note that a low stability values indicate good stability. The TPM is an estimate of the abundance of the microRNA across the two groups.

Gene Ontology Enrichment Analysis

GO enrichment analysis attempts to identify GO terms that are significantly associated with differentially expressed microRNAs. Using miRSearch, we map the differentially expressed microRNAs identified above to their target genes and it is then possible to investigate whether specific GO terms are more likely to be associated with these microRNAs. Two different statistical tests are used and compared. Firstly a standard Fisher's test is used to investigate enrichment of terms between the two test groups. Secondly, the 'Elim' method takes a more conservative approach by incorporating the topology of the GO network to compensate for local dependencies between GO which can mask significant GO terms. Comparisons of the predictions from these two methods can highlight truly relevant GO terms.

The figure below shows a comparison of the results for the GO (Biological process) terms associated with the significantly differentially expressed microRNAs that were identified between groups; normal and FAP_patient. Complete GO enrichment analysis for all of the comparisons is presented in the associated GO folder in the full dataset supplied with the report. The Cellular component (CC) and Molecular functions (MF) analysis are presented in the associated data folder.

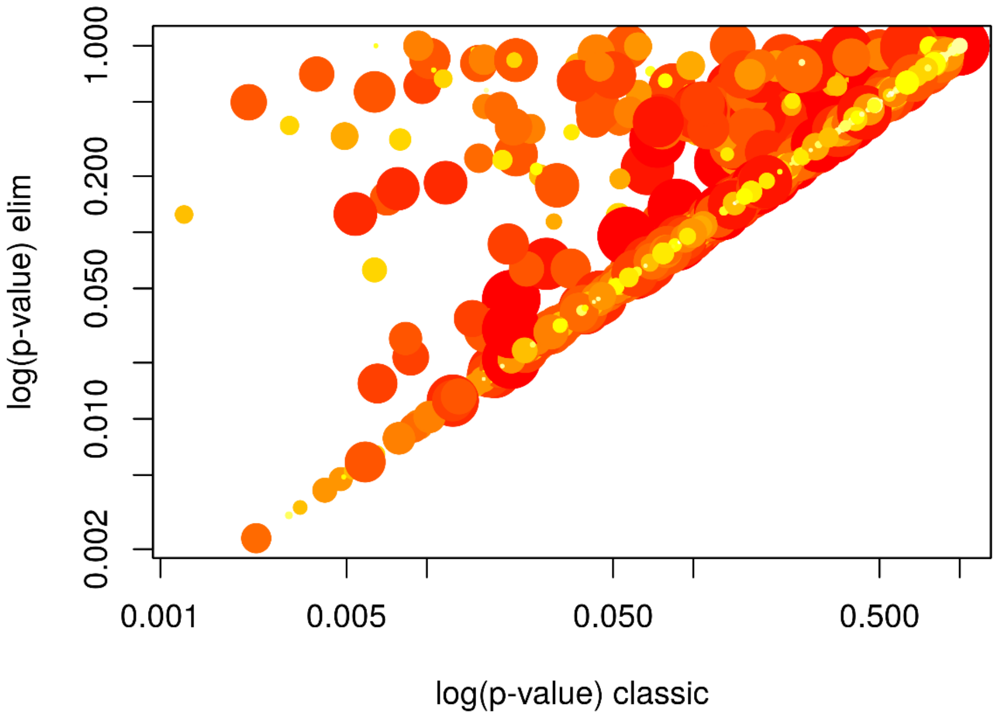

Figure 14. Scatter plot for significantly enriched GO terms predicted to be associated with differentially expressed genes. Plot shows a comparison of the results obtained by the two statistical tests used. Values along diagonal are consistent between both methods with values in the bottom left of the plot corresponding to the terms with most reliable estimates from both methods. Size of dot is proportional to number of genes mapping to that GO term and coloring represents number of significantly differentially expressed genes corresponding to that term with dark red representing more genes and yellow representing fewer.

A list of potentially significant GO (Biological process) terms is given in the table below.

GOID	Term	P-value
GO:0045666	positive regulation of neuron differentiation	0.0023
GO:0045907	positive regulation of vasoconstriction	0.003
GO:0071695	anatomical structure maturation	0.0033
GO:2001237	negative regulation of extrinsic apoptotic signaling pathway	0.0041
GO:0060761	negative regulation of response to cytokine stimulus	0.0043
GO:0030307	positive regulation of cell growth	0.0047
GO:2000727	positive regulation of cardiac muscle cell differentiation	0.0049
GO:1903510	mucopolysaccharide metabolic process	0.0053
GO:2000145	regulation of cell motility	0.0059
GO:0002246	wound healing involved in inflammatory response	0.0064
GO:0030516	regulation of axon extension	0.0065
GO:0021549	cerebellum development	0.0076
GO:0007405	neuroblast proliferation	0.0079
GO:0044089	positive regulation of cellular component biogenesis	0.0079
GO:0043491	protein kinase B signaling	0.008
GO:0036089	cleavage furrow formation	0.008
GO:0035265	organ growth	0.0089
GO:0010631	epithelial cell migration	0.0093
GO:0030004	cellular monovalent inorganic cation homeostasis	0.0094
GO:0031330	negative regulation of cellular catabolic process	0.0095

Table 7. The significant GO terms for the genes targets of microRNAs found to be differentially expressed between normal and FAP_patient and their corresponding annotation for Biological process (BP). The associated network topology is shown in figure 13.

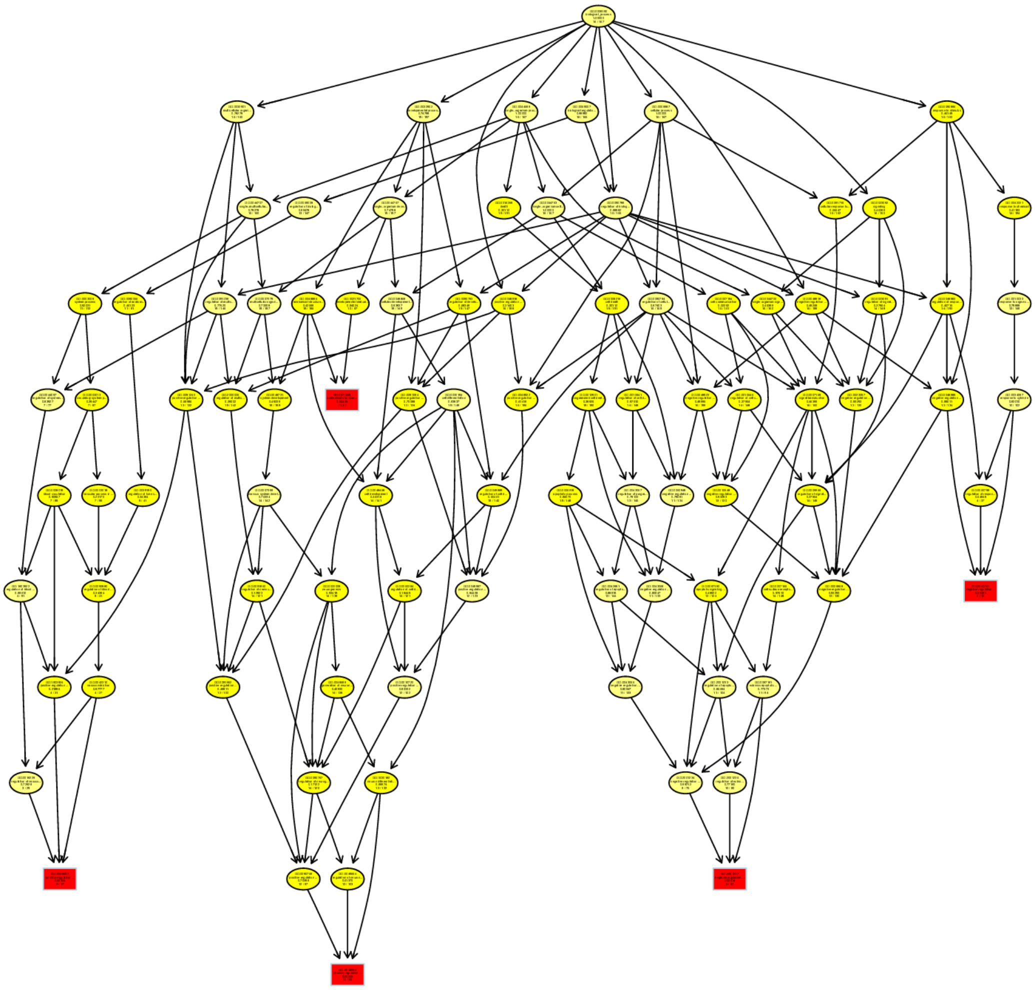

Figure 15. GO network generated from the GO terms predicted to be enriched for the Biological process (BP vocabulary). Nodes are colored from red to yellow with the node with the strongest support colored red and nodes with no significant enrichment colored yellow. The five nodes with strongest support are marked with rectangular nodes. A high-resolution version of this graph is found in the supplementary Figures.

All comparisons

Only GO results for one process and one comparison is presented in the report as an example. For a full list of processes and comparisons see the file Data_Ref_5223.xlsx. The file contains all the comparisons results in separate sheets (tabs)

Identification of novel microRNAs previously reported in other organisms

Following identification of known microRNAs, as defined by miRBase for the host organism, we subsequently searched for potential novel microRNAs in your samples. The objective of this analysis is to identify new microRNAs – this is particularly important for organisms that are less well studied. We use a two-step analysis and each step is described below.

Step 1. Search other organisms in miRBase

Identify matches between the sequenced reads in your samples and the sequences of known microRNAs in miRBase for other organisms.

The motivation behind this analysis is to leverage the microRNA research conducted for other organisms. Due to their functional properties it is likely that at least some microRNAs are under a selective pressure, which would impose conservation between related organisms. This is especially important when working with organisms that are poorly annotated in miRBase.

Example:

Let's consider an experiment conducted in *Bos taurus*. In this experiment significant quantities of the sequence 5'-auacacauacacgcaacacacau-3' was identified. In miRBase this sequence does not match any known *Bos taurus* microRNAs.
This sequence, however, is identical to the human microRNA *hsa-miR-466* and we would consequently report the sequence as a possible new microRNA in *Bos taurus.*

We will report these microRNAs with their known name, e.g. *hsa-miR-466* in the example above. If the sequence has multiple names in miRBase, which is the case for microRNAs identified in multiple organisms, we report only one of the names.

Step 2. miRPara prediction software

Identify sequences which could be novel microRNAs that are not known in any organism annotated in miRBase.
The motivation behind this analysis is to identify truly novel microRNAs based on the structural properties of the genomic regions in which these sequences align.

This analysis is based on the machine learning algorithm implemented in the miRPara software (Wu et al., 2011).

The following two sections contain the results from this investigation. The first section contains the result from the search in miRBase. The second section contains the results from the miRPara prediction software.

Below is a list of miRNA detected in the sequencing data reported in other species than hsa in mirBase sorted by chromosome. For a complete list refer to the Data_ref_5223.xlsx.

Name	Chr	Strand	Start	Stop	Sequence
cfa-miR-142	14	+	70706828	70706844	CCCATAAAGTAGAAAGCACTA

Table 8. The microRNAs identified by sequence homology to microRNAs in miRBase. See full list of homologous microRNAs found in miRBase in Excel file Data_Ref_5061.xlsx.

Identification of novel microRNAs

Putative novel microRNAs are predicted from the sequences that do not map to any organism found in miRbase, or to other known RNA sequences. miRPara (Wu *et al., 2011)* is used to analyze the potential folding of these sequences. These results are combined to identify putative novel microRNAs.

The table below shows a summary of the differential expression analysis for the microRNA prediction.

Id	Chr	Strand	Start	Stop	Sequence
put-miR-1	11	-	44784480	44784503	GTTTCCGTAGTGTAGTGGTTATCA
put-miR-2	16	+	87887509	87887533	CTGTCACGTCTGCGGCTGTCACGTC
put-miR-3	10	+	1095230	1095253	TCAGACGTCTCGAGGCTCGCGTTC
put-miR-4	4	+	109278031	109278049	TGATGTAGTAGGTTGTGTT
put-miR-5	5	+	122990815	122990832	CATGGACGGTGTGAGGCT
put-miR-6	7	+	18159277	18159293	AATCTGACTGTCTAATT
put-miR-7	4	+	107531609	107531625	ACCCACTCTGATCACCA
put-miR-8	17	+	71874183	71874200	AGAGGGACGGCCGGGGGT
put-miR-9	7	+	2102746	2102765	ACACTGGGACTGAGACACGG
put-miR-10	20	+	18309660	18309682	ATGGTAGTGGGTTATCAGAACTT
put-miR-11	5	+	141229349	141229372	TCTCTGTTTTCCGACCTCTCCGGA
put-miR-12	17	+	75675457	75675479	TAGCACCTGCCGAGCACTGAGAA
put-miR-13	1	+	234973730	234973752	ATGGTAGTGGGTTATCAGAACTT
put-miR-14	22	+	50629606	50629628	CCCGGCTGTCCAAGAAGAGGGCA
put-miR-15	12	+	96666261	96666277	GAGAAACGGCTGGAGAG
put-miR-16	10	+	71982317	71982343	GCATTGGTGGTTCAGGGGTAGAATTCT
put-miR-17	8	-	120083247	120083269	ACTGGACTTGGAGCCAGAAGGCC
put-miR-18	11	+	127327252	127327269	GTCTTGCCTCTGTCACCA
put-miR-19	7	+	143079664	143079686	GCTCTGACCTCTGACCCTCTAGC
put-miR-20	1	+	10782719	10782735	TTCGGACTGGCCCAGGG

Table 9. The first 20 predicted microRNAs identified based on counts and secondary structure according to miRPara classification score. See full list of predicted microRNAs in Excel file Data_Ref_5223.xlsx.

Conclusion and next steps

microRNA Next Generation Sequencing libraries were successfully prepared, quantified and sequenced for all your samples.. The data passed all QC metrics, with high Q-score, indicating good technical performance of the NGS experiment. A high percentage of the reads could be mapped to the reference genome, indicating that the samples were of high quality.

The large 30 nt insert peak seen in the library distribution plot includes many reads derived from Y RNAs and their pseudogenes. Note also that there are probably also un-annotated potential pseudogenes found in the reference genome. Previously, 5' Y RNA fragments have been reported as a major contributor to the smallRNA content in Serum and Plasma (Dhahbi J.M., et al. 2013).

The unsupervised analysis do not cluster the samples in concordance with the biological groups, indicating that other factors are major contributors to the overall variation seen in the microRNA profiles.

Most of the comparisons performed are with group sizes n<3, which do not allow for assessment of the statistical significance of the observations. Hence differential expression for these comparisons can only be evaluated based on fold change. Only for set1 (control vs. FAP) can be p-values be calculated.

The microRNA prediction found predicted microRNAs in your dataset based on putative precursor hairpin structures and these were included in the subsequent supervised differential expression analysis.

Note, when navigating through the data, counts lower than 10-15 TPM (counts per million of mapped reads, on average) per group might be difficult to validate in a subsequent qPCR experiment.

We would like to help you interpret the data presented in this report and guide you on how best to proceed with subsequent experiments. If you would like to arrange a time to discuss the data with us in more detail, please do not hesitate to contact DxServices@exiqon.com and we will be happy to arrange a phone call with you.

Online search tool - miRSearch

miRSearch is found at https://www.exiqon.com/mirsearch and is an online search tool which quickly finds and displays microRNAs relevant for your research as well as detailed information about each microRNA in the most recent version of miRBase.

Search using a keyword or gene name and find:

microRNAs associated with specific diseases

microRNAs found in specific tissues/samples

microRNAs that regulate specific genes (validated and predicted interactions)

Search using a microRNA name and find:

Regulated genes (validated and predicted interactions)

Potentially co-transcribed microRNAs

Diseases in which the microRNA has been shown to be regulated

Tissues/samples in which the microRNA has been found

Validated targets as well as diseases and tissue/sample information is supported by references with links to PubMed.

Predicted interactions can be viewed graphically even down to sequence level.

When microRNAs are reported to target gene transcripts in the literature, different ways of annotating the target are used depending on the journal. miRSearch uses an advanced algorithm to cross-reference all these annotations so that a comprehensive list of microRNA-mRNA interactions can be displayed. Please note that the most recently validated microRNAs may not be displayed.

miRSearch will also find microRNAs predicted to bind to your target sequence. These results are based on TargetScan 1 predictions and scored so that it is easy to see the relative importance of the microRNAs. Predicted microRNAs have not been experimentally validated.

Finally, miRSearch links to three different product types for analysis of microRNAs relevant for your research: qPCR assays, inhibitors and detection probes for in situ hybridization or Northern blot.

Materials and methods

All experiments were conducted at Exiqon Services, Denmark.

Library preparation and Next Generation sequencing

RNA was isolated from 500µl plasma with proprietary RNA isolation protocol optimized for serum/plasma (no carrier added). Total RNA was eluted in ultra-low volume.

The library preparation was done using the NEBNext® Small RNA Library preparation kit (New England Biolabs). A total of 6µl of total RNA was converted into microRNA NGS libraries. Adapters were ligated to the RNA. Then RNA was converted to cDNA. The cDNA was amplified using PCR (18 cycles) and during the PCR indices were added. After PCR the samples were purified. Library preparation QC was performed using either Bioanalyzer 2100 (Agilent) or TapeStation 4200 (Agilent). Based on quality of the inserts and the concentration measurements the libraries were pooled in equimolar ratios. The pool was then size selected using the LabChip XT (PerkinElmer) aiming to select the fraction with the size corresponding to microRNA libraries (~ 145 nt). The library pool(s) were quantified using the qPCR KAPA Library Quantification Kit (KAPA Biosystems). The library pool were then sequenced on a Nextseq500 sequencing instrument according to the manufacturer instructions. Raw data was de-multiplexed and FASTQ files for each sample were generated using the bcl2fastq software (Illumina inc.). FASTQ data were checked using the FastQC tool (http://www.bioinformatics.babraham.ac.uk/projects/fastqc/). FASTQ file quality scores uses quality score binning enabling a more compact storage of raw sequences. Using only 8 levels (Levels: No call, 6, 15, 22, 27, 33, 37, 40) of quality the method has been tested and found to virtually loss-less. Please refer to Illumina white paper for further explanation of the quality score binning system.
(http://res.illumina.com/documents/products/whitepapers/whitepaper_datacompression.pdf).

Validating your NGS results

The following section is meant as a guideline for selecting the relevant microRNAs from your NGS study to be validated with qPCR. In order to be successful in this validation it is important to follow a set of criteria for selecting candidate microRNAs for validation.

Fold change

It is recommended to identify microRNAs that show a sufficient level of regulation across the relevant groups of samples in the study. While it is possible to validate microRNAs that show small regulations it is important to remember that smaller fold changes tend to be relatively more affected by technical variance. Such changes are thus associated with increased risk of false-positive signals. Based on this we recommend to include microRNAs showing more than a 2-fold up- or down-regulation in a validation study by qPCR (corresponding to +/- 1.0 logFoldChange). To study microRNAs with small fold changes, considerably more technical or biological replicates should be included in the validation.

Statistical significance

For studies where at least three replicates have been supplied for each group, the present NGS includes p-values. These should be used to evaluate the significance of the findings. In the sheets named Data_ref_5223.xlsx, the p-values and possibly adjusted p-values are listed for the performed comparisons. For the adjusted p-values, values <0.05 should be considered as significant, and microRNAs showing a statistically significant regulation have a greater chance of validating by qPCR.

Number of reads

It is important to consider the read counts of the microRNAs of interest when designing a validation experiment. Internal microRNA NGS data has shown that microRNAs with low read count can be hard to validate.

An example of this is seen in the figure on the next page comparing microRNA total reads in a 4 million read NGS experiment (Total human brain sample (Ambion)). Generally, after a total read number of 50 is reached, successful qPCR can be done (Cq < 37). We use Cq values of 37 as a cutoff to ensure robust and reproducible data points. Consider this when selecting assays for validation. Note however these are averages and affected by tissue type and the microRNA read number as well as microRNA numbers and distribution within a given sample. Exiqon Service Scientists can help you design the optimal experimental setup to maximize your likelihood of success.

What to look for?

“The important criteria to consider when designing a validation experiment are the microRNA fold change, statistical significance, number of counts, and reference microRNA.”

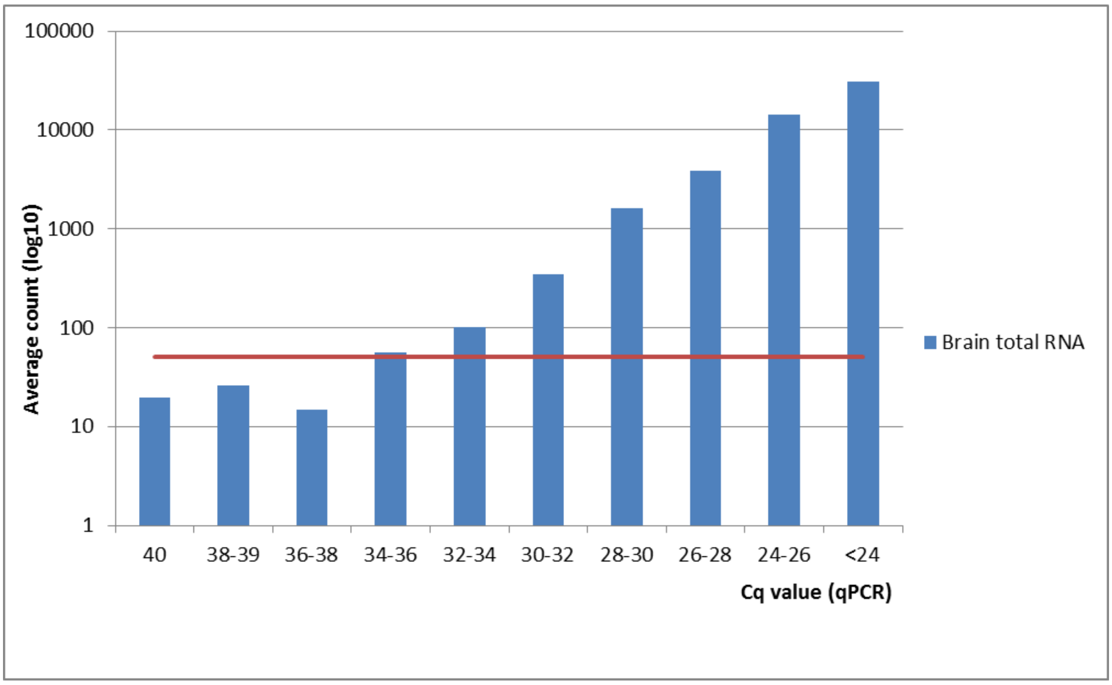

Figure 16. Comparison of NGS data and miRCURY LNA™ Universal RT microRNA PCR data (Human panel I+II). microRNAs with counts above the red line have good chance of being validated using Exiqon's qPCR platform. At low counts it is very difficult to get consistent signal on a qPCR platform. However, microRNAs with approximately 25 tags per million reads (TPM) or above will most likely give a robust signal upon qPCR validation. 25 TPM corresponds to Cq values of approximately 35. Note however these are averages and affected by tissue type and the microRNA number and distribution within the sample.

Selecting reference genes for qPCR normalization

In general, we recommend that the endogenous reference genes used should be stably expressed microRNAs rather than longer RNA species such as snoRNAs and snRNAs including U6. This is because microRNAs are so short that they may have very different behaviour during extraction and reverse transcription compared to longer transcripts (Vandesompele et al., 2002). The NGS experiment may be able to provide you with information on which microRNAs that could serve well as reference genes for normalization in qPCR. The general idea is to identify microRNAs which show a very stable (constant) expression across the different samples in the study. To assist you in identifying such stably expressed genes, we have included a NormFinder analysis (www.mdl.dk) in this report.

Pick-&-Mix – Design your own microRNA qPCR panels for validation

Exiqon's miRCURY LNA™ Universal RT microRNA PCR system offers unmatched sensitivity and specificity coupled with the convenience of a single universal reverse transcription step. Profile hundreds of microRNAs on panels using just 20ng total RNA without the need of pre-amplification. The whole process takes just 3 hours. Exiqon offers custom Pick-&-Mix microRNA PCR Panels. Design your own 96- or 384-well microRNA qPCR plates based on a fully flexible layout and several conveniently pre-defined layouts. Place miRCURY LNA™ Universal RT microRNA PCR assays or your own custom LNA™-enhanced assays onto the panel using an intuitive and easy-to-use online plate configurator. The panels are delivered ready-to-use containing one single assay per well for a 10 µL reaction volume - just add cDNA

and ExiLENT SYBR® Green master mix. Read more here:
http://www.exiqon.com/pick-and-mix

If you prefer, Exiqon Services can also run these experiments for you. Our state-of-the-art laboratories use high throughput robotic pipetting stations that ensure superior reproducibility. All provided data undergo rigorous quality control to ensure that the data is based on the correct PCR product. If you chose this option, we will provide you with raw and normalized Cq-values, the relevant statistical comparisons as well as thorough consultation and full data report.

References

Andersen, C.et al. (2004) Normalization of Real-Time Quantitative Reverse Transcription-PCR Data: A Model-Based Variance Estimation Approach to Identify Genes Suited for Normalization, Applied to Bladder and Colon Cancer Data Sets. Cancer Res., 64, 5245-5250.

Benjamini and Hochberg (1995) Controlling the False Discovery Rate: A Practical and Powerful Approach to Multiple Testing. Journal of the Royal Statistical Society Series B, 57, 289-300.

Cock PJA., et al (2010) The Sanger FASTQ file format for sequences with quality scores, and the Solexa/Illumina FASTQ variants. Nucleic Acids Res. 2010 Apr; 38(6): 1767–1771.

Dhahbi J.M., et al. (2013) 5'-Y RNA fragments derived by processing of transcripts from specific YRNA genes and pseudogenes are abundant in human serum and plasma. *Physiol Genomics* 45: 990–998.

Metpally R.P.R. et al. (2012) Comparison of Analysis Tools for miRNA High Throughput Sequencing Using Nerve Crush as a Model. Front Genet. 4(20), 1-13.

Robinson MD, Smyth GK (2007) Moderated statistical tests for assessing differences in tag abundance. Bioinformatics, 23(21):2881-2887.

Robinson and Oshlack, (2010) A scaling normalization method for differential expression analysis of RNA-seq data.

Wu Y., et al. (2011) MiRPara: a SVM-based software tool for prediction of most probable microRNA coding regions in genome scale sequences. BMC Bioinformatics, 12(1):107.

The Gene Ontology Consortium (2000) Gene ontology: tool for the unification of biology. Nat. Genet,25(1):25-9.

Frequently asked questions

What is "Q-score"?

Answer: A quality score (or Q-score) is a prediction of the probability of an incorrect base call. Q-score = -10 log10(P(~X)) where P(~X) is the estimated probability of the base call being wrong. A quality score of 10 indicates an error probability of 0.1, a quality score of 20 indicates an error probability of 0.01, a quality score of 30 indicates an error probability of 0.001, and so on.

What is chastity filtering?

Answer: This filter is applied by the on-board instrument computer, and is used to identify clusters with a low signal to noise ratio, often as a result of two adjacent clusters being so physically close together that their signals cannot be measured independently. All reads (both failing and passing the filter) are included in the fastq file, and the filter information is encoded in the id for each read.

Which data points were removed due to data quality controls?

Answer: Data for analysis needs to pass the following criteria:

- High quality score (Q score) and read length of >15.
- Be mappable to the corresponding genome(s) and/or databases.
- Pass background filtering based on read numbers (to remove low copy reads).

If the data points do not meet these criteria, they are removed from the dataset.

What is the difference between "reads" and "counts"?

Answer: "Reads" refer to anything detected. "Counts" describe reads that align to a position in the reference genome.

How are novel microRNA species identified?

Answer: Novel microRNAs are predicted based on read counts (mirDeep) and sequence composition and structure (miRPara). By considering these two distinct perspectives we can make more robust predictions for novel microRNAs.

What is GO analysis?

Answer: The Gene Ontology is a formal representation of species independent knowledge associated with genes and their products. This means the information can be parsed and analyzed by computers to associate the knowledge with the results from biological experiments in order to gain further insight.

What is multiple testing corrections?

Answer: When a large number of statistical tests are carried out simultaneously, ordinary p-values need to be adjusted in order to control the number of false positives, i.e. the number of genes for which the null hypothesis 'the gene is equally expressed between groups' is incorrectly rejected – type I errors (Benjamini and Hochberg, 1995).

Is it possible to expand the groups later?

Answer: Yes, it is possible to add more samples to groups at a later date. Additional bioinformatics analyses will be requested as custom service.

The product offering says 7.5 million reads. However when looking at my data I see fewer mapping to microRNAs

Answer: We generate 7.5 million passed filter reads for each sample. During the size selection of the library preparation we collect the fraction corresponding to the size range of the microRNA. But in this fraction other RNA species can be found as well, most likely as results of degraded rRNA or tRNA. Normally around 10-60% of the reads are mappable to microRNA depending on the quality and type of sample.

Appendix 1 - Pipeline analysis overview

The analysis pipeline combines several steps for QC of sequencing data to mapping and statistical analysis. Please refer to the different part of the summary report for an in-depth explanation of the processes and the results.

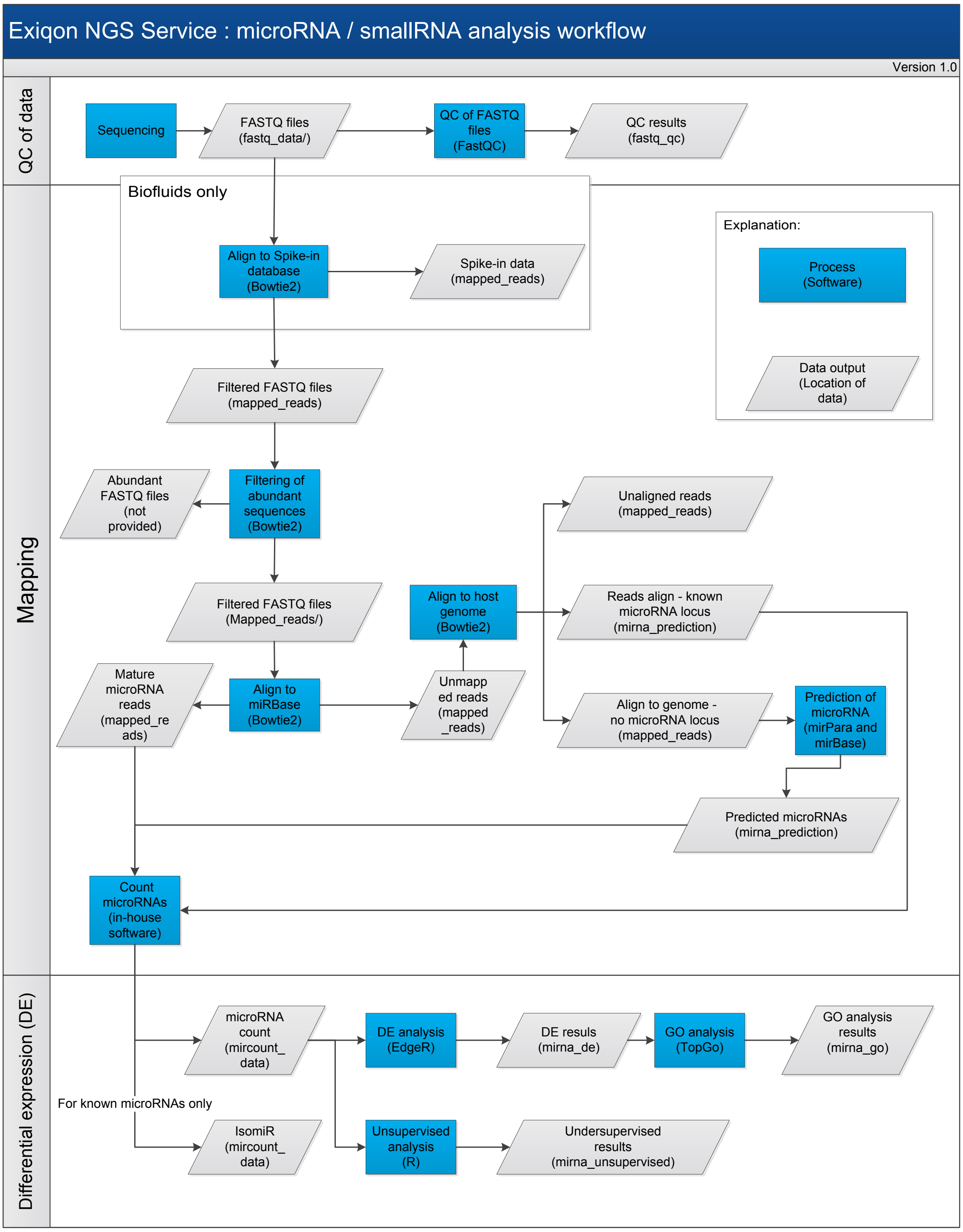

Figure 17 Overview of the analysis pipeline for microRNA and smallRNA projects. Blue square boxes indicates a process, the software/tool performing the process is specified in parenthesis. Grey parallelogram boxes indicates a data output and the output location is specified in parenthesis. Note that the workflow contains an extra step if the samples originate from biofluids and spike-ins have been added to monitor the extraction. Refer to the summary report (including references) for further explanation of each step. Also refer to the file_description.html file for details about the data outputs, how to browse the files and how to interpret the results
