# Supplementary material for: Assessment of circulating microRNA specific for patients with familial adenomatous polyposis
Source: PLoS One. 2021 May 4;16(5):e0250072. doi: 10.1371/journal.pone.0250072 (PMC8096076; doi:10.1371/journal.pone.0250072)
Supplement: Attachment — Submitted filename: Response to Reviewers.docx [file pone.0250072.s004.docx]

Dear Reviewers,

Thank you for your time for reviewing the manuscript and describing the useful comments to us. We have revised the manuscript and responded to your comments as possible as we could.

We apologized to the reviewers for not performing the appropriate basic experiments to support our findings, although we agree the importance of the further experiments. We considered that new samples from the FAP patients or APC deficient mice were most suitable for the experiments. However, performing the experiments is beyond our capacity now. Then, we added some data to support our findings and mainly revised Abstract and Discussion to clearly describe the findings and problems of our study.

The association between plasma miR-143-3p and pathological T factor / lymph node metastasis and the rarity of mir-143-3p upregulation in colonic tumors were added in the revised manuscript.

Discussion was organized by circulating miR-143-3p, tumor miR-143-3p, inconsistent expression between plasma and tumor, target of mir-143-3p, speculation, and limitation of the study, because these contents were described roughly in the first version.

**Reviewer #1:**

*“Assessment of circulating microRNA specific for patients with familial adenomatous polyposis” by Yamano and colleagues is an effort to downselect a cohort of microRNAs from a group of clinical samples obtained from 15 familial adenomatous polyposis and 7 controls. Using next generation sequencing the authors identify miRs that may be potential biomarkers for pathology.*

*While the study is strengthened by the observation of clinical findings, the lack of any functional studies diminishes the enthusiasm in the study and the potential of the findings. In order for the study to be improved the following sets of data are highly encouraged to be introduced.*

1. *Gain and loss of functional studies using selected microRNAs. Judicious choice of controls are warranted to strengthen the observations.*

Thank you for your comment. Many studies using cancer cell lines have already confirmed onco-suppressive effect of mir-143-3p. Our data showed that circulating mir-143 was upregulated in FAP patients, although it seemed no significant influence on tumorigenicity by upregulated miR-143-3p. Then, we considered that basic studies using colonic samples from FAP patients or APC deficient mice may be more suitable for functional study than cancer cell lines. However, these experiments were beyond our capacity now.

Instead of functional studies, we added the data regarding association between plasma mir-143-3p and pathological data (pathological T factor and lymph node metastasis) in Results. We also assessed tendency of miR-143-3p expression in colonic tumors and described in Results. We also revised Discussion.

**Results**

**Validation**

(First version) Line: 176-178

There was no significant difference in miR-143-3p expression based on patient sex (*P* = 0.29), levels before or after radical surgery (*P* = 0.43), the existence of desmoid tumor (*P* = 0.33), or family history (*P* =0.91).

(Revised version) Line200-204

There was no significant difference in miR-143-3p expression based on pathological T factor (*P* = 0.25, Fig. 2B), pathological node positive or not (*P* =0.42), patient sex (*P* = 0.29), levels before or after radical surgery (*P* = 0.43), the existence of desmoid tumor (*P* = 0.33), or family history (*P* =0.91).

**MiR-143-3p expression in colonic tumors**

(First version) No description.

(Revised version) Line:216-217

Then, upregulation was significantly rare by chi-square test (*P* =0.02).

**Discussion**

(First version) Line215-217

Our study had several limitations. First, our results regarding the upregulation of circulating miR-143 and downregulation of colonic tumor miR-143 has not been confirmed in studies using APC-deficient mice.

(Revised version) Line: 257-260

Our study had several limitations mainly because we have described the data from clinical samples, which were not supported by basic experiments.

First, biological effect of miR-143-3p have not been evaluated by using colonic samples derived from FAP patients or APC-deficient mice.

2. *What are the targets being regulated by the microRNAs of interests and how are they affecting the disease process?*

Thank you for your comment. Target genes of mir-143-3p against cancer cell lines have been reported already as we described in Discussion. In this study, upregulated mir-143-3p in FAP patients seemed ineffective to prevent tumorigenesis. Then, the specimens from FAP patients or APC deficient mice may be more preferable than cancer cell lines to assess the target genes. However, these experiments were beyond our capacity.

The source cells of upregulated miR-143-3p expression are also critical to explain our findings. We have added the sentence in revised Discussion as below.

**Discussion**

(First version) Line194-196

There are many target genes of the tumor suppressor function of miR-143 in CRC, including *Bcl-2, CASP3*, *CD44, KLF5, KRAS, BRAF*, *TLR2*, *SOD1*, *IGF1R*, *ITGA6,* and *ASAP3* [14-19].

(Revised version) Line:249-252

There are many target genes of the tumor suppressor function of miR-143 in CRC, including *Bcl-2, CASP3*, *CD44, KLF5, KRAS, BRAF*, *TLR2*, *SOD1*, *IGF1R*, *ITGA6,* and *ASAP3* [27-32]. However, these results were performed using the cancer cell lines, but not benign tissues or cells from FAP patients or APC deficient mice.

3. *Experimental evidence to highlight the biological/ physiological importance of occurrence of miRs in biosamples such as plasma and tumors- and also if there is an occurrence at 1 site, do they lose the potential to be biomarkers?*

The reason why mir-143-3p showed different expression pattern by the location was undetermined. The critical issue should be the cellular source of plasma mir-143-3p, which is also elevated in the patients with cerebral atherosclerosis, acute ischemic stroke, and amyotrophic lateral sclerosis. Mesenchymal cells such as fibroblasts and smooth muscle cells have been reported to highly express mir-143-3p compared to colonic epithelial cells. Well-organized experiments are necessary for understanding the mechanisms of mir-143-3p in human.

We added the sentences in Discussion as below.

**Discussion**

(First version) Line:196-197

However, our current results suggested the upregulation of plasma miR-143-3p was insufficient for inhibiting tumor progression in patients with FAP.

(Revised version) Line:245-248

Then, our current results were inconsistent with the previous studies because upregulation of plasma miR-143-3p seemed insufficient for inhibiting tumor progression in FAP patients. Difference of miR-143-3p expression by sample location and its interaction has not been evaluated.

(First version) Line:212-214

Our finding that suggests that the upregulation of mir-143-3p in colonic tumor is associated with desmoid tumor should be validated by further studies. Desmoid tumor is considered a serious neoplasm related with FAP and difficult to diagnose [32].

(Revised version) Line: 261-264

Second, the sources of upregulated plasma miR-143-3p have not been confirmed. Possible sources of mir-143 could be mesenchymal cells such as fibroblast and smooth muscle cells [34]. Then, higher expression of miR-143-3p in colonic tumor with desmoid tumor (fibromatosis) in our study seemed to be associated with this speculation. [35].

**Reviewer #2**:

*In this manuscript by Yamano et al, the authors are looking for circulating miRNA in patients with a hereditary disease called familial adenomatous polyposis. Both plasma and tissue miRNA expression was tested from patients with FAP vs control (tumor or mucosa for tissue).*

1. *The authors provide no rationale for performing the experiments. The expression of APC is used for FAP, and given that this is a hereditary disease, there is often family history present as well. The authors make no argument to the need of another marker for FAP. They describe in the introduction that they are going to compare 3 groups: healthy vs. FAP vs. CRC, and that potentially they could find a marker that would span both FAP and non-hereditary CRC. This wasn't done.*

We hypothesized that microRNA profiling in genetic diseases should be modified to compensate the disadvantages induced by genetic abnormality. Then, we considered that FAP patients should have the specific profiling of circulating microRNAs to compensate the influence by loss of APC function. We selected FAP as a representative genetic disease among gastrointestinal diseases, because we have constantly experienced FAP patients and published manuscripts for several decades. Another representative genetic disease, Lynch syndrome, has variable clinical and genetic features than FAP.

Most FAP patients are usually diagnosed by clinical features such as colonic polyposis and family history without confirming APC mutation. Diagnosis of Lynch syndrome is more difficult than that of FAP , and CRC patients are not routinely assessed for Lynch syndrome. We expected that effective diagnosis by circulating microRNAs for FAP could be applied for Lynch.

We apologize that description is so insufficient that you misunderstand the study. The description showed our hypothesis that circulating microRNAs profiling maybe specific for FAP patients, which should be different from healthy patients and sporadic CRC patients. We have not assessed blood samples derived from sporadic CRC patients.

We have revised the sentences as below. And added reference 10 regarding FAP and reference 13 regarding Lynch syndrome.

**Abstract**

(First version) Line: 34-36

We assessed circulating miRNAs of patients with familial adenomatous polyposis (FAP) as a hereditary disease and prototype of colorectal cancer progression.

(Revised version) Line:28-31

We assessed circulating miRNAs of patients with familial adenomatous polyposis (FAP) as a hereditary disease and prototype of colorectal cancer progression to evaluate as possible biomarkers of hereditary gastrointestinal diseases.

**Introduction**

(First version) No description

(Revised version) Line: 64-65

We have experienced hundreds of FAP patients for several decades [10].

(First version) Line:77-80

Therefore, we chose FAP as a representative hereditary disease and a prototype of CRC and attempted to identify and assess a plasma miRNA specific for patients with FAP as a potential biomarker.

(Revised version) Line:71-77

Although most FAP cases are diagnosed by endoscopic examination without further genetic test, application of endoscopic examination for younger family is usually difficult to perform. Lynch syndrome, another hereditary disease, is difficult for diagnosis by endoscopic examination alone, and needs further examinations including genetic test [13]. Therefore, we chose FAP as a representative hereditary disease to evaluate the possibility of circulating miRNA as a potential biomarker of hereditary gastrointestinal diseases.

**Discussion**

(First version) Line:189-191

In the current study, we explored circulating miRNAs specific for patients with FAP and found that miR-143-3p was upregulated in the plasma of patients with FAP, but not in colonic tumors.

(Revised version) Line:231

In the current study, we explored circulating miRNAs specific for FAP patients.

(First version) Line:217-218

Second, we did not validate the usefulness of measuring circulating miR-143 in family members of patients with FAP.

(Revised version) Line: 265-266

Third, we did not validate the usefulness of measuring circulating miR-143-3p in family members of FAP patients, who were not diagnosed with FAP or not.

2. *It's unclear why the authors use two control miRNAs for expression of miR143-3p, but not for any of the other potential candidates.*

Different from tumor, control miRNA for blood samples has not been confirmed, although miR-103-3p is considered as one of the common control miRNAs. Next generation sequence data indicated miR-16-5p was suitable control miRNA, because of similar expression between FAP patients and healthy donors. Then, we assessed these two miRNAs. Mir-103a-3p is one of the internal control miRNAs of blood samples recommended by manufacture’s instruction from Exiqon.

**Abstract**

(First version) Line:39-40

With minimum differences among samples, miR-16-5p was selected as an internal control.

(Revised version) Line:34-35

MiR-16-5p was also assessed as an internal control due to minimum difference in expression across FAP patients and healthy donors.

**Results**

**Exploration of potential plasma biomarkers of FAP**

(First version)

No description in Results.

(Revised version) Line:172-174

NGS also showed that miR-16-5p was most stably expressed across FAP patients and healthy donors, which indicated a good candidate for normalizer in quantitative PCR study.

3. *The Figures are mislabeled. There are only 2 figures (with A & B on both).*

Thank you for your comment. We revised the figure numbers as below.

**Results**

**Exploration of potential plasma biomarkers of FAP**

(First version) Line:158

Fig.1

(Revised version) Line:166

Fig.1A

**Assessment of miRNA expression fluctuations**

(First version) Line:169

Fig. 2

(Revised version) Line:166

Fig.1B

**Validation**

(First version) Line: 175

Fig. 3

(Revised version) Line:200

Fig 2A

(First version) No description

(Revised version) Line:202

Fig 2B

**MiR-143-3p expression in colonic tumors**

(First version) Line: 181

Fig. 2B

(Revised version) Line:219

Fig 3

4. *The expression of miRNA in the tumors is mostly decreased compared to control tissue, but there is no significant change. No explanation is provided for the difference between plasma and tumor tissue.*

We statistically evaluated mir-143-3p expression in colonic tumors.

**Material and methods**

**Statistical analysis**

(First version) No description

(Revised version) Line:160-161

The proportion of category was assessed by chi-square test.

**Results**

**MiR-143-3p expression in colonic tumors**

(First version) Line: 180-182

The expression of miR-143-3p in the colonic tumors was upregulated with fold change more than two in one patient (Table 3, Figure 2B). Nine patients exhibited less than half expression in normal mucosa compared to the matched tumor samples.

(Revised version) Line:214-217

The expression of miR-143-3p in the colonic tumors was upregulated with fold change more than two in one patient alone (Table 3). Nine patients exhibited less than half expression in normal mucosa compared to the matched tumor samples. Then, upregulation was significantly rare by chi-square test (*P* =0.02).

**Discussion**

(First version) Line: 189-191

In the current study, we explored circulating miRNAs specific for patients with FAP and found that miR-143-3p was upregulated in the plasma of patients with FAP, but not in colonic tumors.

(Revised version) Line:231-232

We found that miR-143-3p was upregulated in plasma, but not in colonic tumors.

(First version) Line: 196-197

However, our current results suggested the upregulation of plasma miR-143-3p was insufficient for inhibiting tumor progression in patients with FAP.

(Revised version) Line:243-248

Rarity of miR-143-3p upregulation in colonic tumors of FAP patients is consistent with a previous report [26].

Then, our current results were inconsistent with the previous studies because upregulation of plasma miR-143-3p seemed insufficient for inhibiting tumor progression in FAP patients. Difference of miR-143-3p expression by sample location and its interaction has not been evaluated.

*Overall, the major concern with this manuscript is the lack of rationale for the studies and the lack of description of the results.*

Thank you for your comment. We revised introduction and discussion and added the data.

Our rationale is described in Introduction as below.

**Introduction**

Revised version, Line:71-77

Although most FAP cases are diagnosed by endoscopic examination without further genetic test, application of endoscopic examination for younger family is usually difficult to perform. Lynch syndrome, another hereditary disease, is difficult for diagnosis by endoscopic examination alone, and needs further examinations including genetic test [13]. Therefore, we chose FAP as a representative hereditary disease to evaluate the possibility of circulating miRNA as a potential biomarker of hereditary gastrointestinal diseases.

Description of the results were described as below.

**Discussion**

(First version) Line: 189-191

In the current study, we explored circulating miRNAs specific for patients with FAP and found that miR-143-3p was upregulated in the plasma of patients with FAP, but not in colonic tumors.

(First version) Line: 203-207

Circulating miRNAs are promising biomarkers for the diagnosis of CRC [21–23]. However, only a few reports have considered miR-143 as a biomarker of CRC [24–26]. On the other hand, miR-143 has been reported as a candidate biomarker for other diseases, including acute ischemic stroke, cerebral atherosclerosis, pituitary adenoma, and ulcerative colitis [27–30].

(Revised version) Line231-237

We found that miR-143-3p was upregulated in plasma, but not in colonic tumors.

Circulating miRNAs are promising biomarkers for the diagnosis of CRC [15-17]. However, only a few reports have considered miR-143 as a biomarker of CRC [18-20]. On the other hand, circulating miR-143 has been upregulated in other diseases including acute ischemic stroke, cerebral atherosclerosis, amyotrophic lateral sclerosis, and pituitary adenoma [21-24].

Reference 24 has been changed from the manuscript about ulcerative colitis to amyotrophic sclerosis, because the former manuscript described regarding colonic tumors, but not blood samples.

(First version) Line:196-197

However, our current results suggested the upregulation of plasma miR-143-3p was insufficient for inhibiting tumor progression in patients with FAP.

(First version) Line:200-202

Our finding of the downregulation of miR-143 expression in colonic tumors of patients with FAP is consistent with a previous report [20].

(Revised version) Line243-248

Rarity of miR-143-3p upregulation in colonic tumors of FAP patients is consistent with a previous report [26].

Then, our current results were inconsistent with the previous studies because upregulation of plasma miR-143-3p seemed insufficient for inhibiting tumor progression in FAP patients. Difference of miR-143-3p expression by sample location and its interaction has not been evaluated.

Dear Reviewer 1 and Reviewer 2

Under reconstruction of the manuscript, we have changed the style of Abstract and remove one sample from analysis. Re-analysis has not changed significance of the data.

1. We have also revised the **abstract** depending on the journal style.

2. We re-analyzed the data of colonic tumors, because the first version included the sample (F20) regarding SBA data, not colonic tumor.

**Abstract**

(First version)

**Background** Circulating microRNAs (miRNAs) are considered promising biomarkers for diagnosis, prognosis, and treatment efficacy of malignancies. We assessed circulating miRNAs of patients with familial adenomatous polyposis (FAP) as a hereditary disease and prototype of colorectal cancer progression.

(Revised version)

Circulating microRNAs (miRNAs) are considered promising biomarkers for diagnosis, prognosis, and treatment efficacy of malignancies. We assessed circulating miRNAs of patients with familial adenomatous polyposis (FAP) as a hereditary disease and prototype of colorectal cancer progression to evaluate as possible biomarkers of hereditary gastrointestinal diseases.

(First version)

**Methods** MiRNAs specific for patients with FAP were identified using next-generation sequencing (NGS). Those that had moderate copy numbers and significant differences among the samples were selected for further evaluation as candidate biomarkers. With minimum differences among samples, miR-16-5p was selected as an internal control. The miRNAs were assessed by real-time PCR for expression ratios between minimum and maximum values at different time points. The miRNAs with the least expression variation were validated in patient plasma and colonic tumors.

**Results** MiR-143-3p, miR-96-5p, miR-183-5p, and miR-885-5p were selected as candidate biomarkers. Mean expression ratios of miR-143-3p/miR-16-5p, miR-143-3p/miR-103-3p, miR-183-5p/miR-16-5p, and miR-885-5p/miR-16-5p were 2.2, 3.4, 5.1, and 4.9, respectively. MiR-143-3p, with lowest variation ratio, was further assessed using specimens from 16 patients with FAP and 7 healthy donors. Plasma miR-143-3p was upregulated in FAP patients compared to healthy donors (P = 0.04), but not significantly influenced by sex, radical surgery, existence of desmoid tumor, or family history. However, miR-143-3p expression was not upregulated in colonic tumors of most patients with FAP, except for those with desmoid tumors.

**Conclusions** Our data suggested miR-143-3p expression differed between plasma and colonic tumors of most patients with FAP. Upregulation of plasma miR-143-3p expression may be helpful in the diagnosis of FAP, although it has no suppressive effect on tumor progression.

(Revised version)

Next-generation sequencing (NGS) indicated that plasma miR-143-3p, miR-183-5p, and miR-885-5p were candidate biomarkers for five FAP patients compared to three healthy donors due to moderate copy number and significant difference. MiR-16-5p was also assessed as an internal control due to minimum difference in expression across FAP patients and healthy donors. Then, plasma samples were further assessed by real-time PCR. Mean ratios of maximum expression and minimum expression were 2.2 for miR-143-3p/miR-16-5p, 3.4 for miR-143-3p/miR-103a-3p, 5.1 for miR-183-5p/miR-16-5p , and 4.9 for miR-885-5p/miR-16-5p by using eight FAP samples collected at different time points. Then, miR-143-3p/16-5p was further assessed using specimens from 16 FAP patients and 7 healthy donors. Plasma miR-143-3p was upregulated in FAP patients compared to healthy donors (P = 0.04), but not significantly influenced by pathological T factor, lymph node metastasis, sex, radical surgery, existence of desmoid tumor, or family history. However, miR-143-3p expression in colonic tumors was rare for upregulation, although there was a significant difference by existence of desmoid tumors. Our data suggested regulation of miR-143-3p expression differed by samples (plasma or colonic tumors) in most FAP patients. Upregulation of plasma miR-143-3p expression may be helpful for diagnosis of FAP, although suppressive effect on tumorigenesis seemed limited in FAP patients.

**Results**

**MiR-143-3p expression in colonic tumors**

(First version) Line-181-186

Nine patients exhibited less than half expression in normal mucosa compared to the matched tumor samples. There was no significant difference in miR-143-3p expression based on sex (*P* = 0.63), pathology of colonic tumor (*P* = 0.81), existence of gastric (*P* = 0.23) or duodenal polyp (*P* = 0.72), or family history (*P* = 0.36). However, there was a significant difference in miR-143-3p expression based on the existence or absence of desmoid tumor (*P* = 0.0002).

(Revised version) Line: 215-221

Eight patients exhibited less than half expression in normal mucosa compared to the matched tumor samples. Then, upregulation was significantly rare by chi-square test (*P* =0.02). There was no significant difference in miR-143-3p expression based on sex (*P* = 0.55), pathological T factor (*P* = 0.78, Fig. 3), existence of gastric (*P* = 0.14) or duodenal polyp (*P* = 0.59), or family history (*P* = 0.43). However, there was a significant difference in miR-143-3p expression based on the existence or absence of desmoid tumor (*P* <0.0001).
