# Supplementary material for: Assessment of circulating microRNA specific for patients with familial adenomatous polyposis
Source: PLoS One. 2021 May 4;16(5):e0250072. doi: 10.1371/journal.pone.0250072 (PMC8096076; doi:10.1371/journal.pone.0250072)
Supplement: Attachment — Submitted filename: Response to Reviewers comments 2021 0214.docx [file pone.0250072.s005.docx]

Dear Reviewers,

Thank you for your time for reviewing the manuscript and describing the useful comments to us again. We have revised the manuscript and responded to your comments as possible as we could.

***Associate Editor:***

*Please address the comments provided by the reviewers. Especially reviewer #1 wants to see functionality via clinical samples or in vitro experiments. Final decision will be made based on the response (additional functional experiments).*

Thank you for giving us to revise the manuscript again. We have performed *in vitro* experiments depending on Reviewer 1 suggestion and also revised the manuscript depending on Reviewer 2 suggestions.

**Reviewer #1:**

*The authors have failed to address the comments raised about functionality. To simply cite other evidence and not generate any new results to address the queries is not enough. Also how is including "pathological T factor, lymph node metastasis" data association with miR of any help to understand miR specific disease function.*

*If the clinical samples were not available to perform the assays asked for the authors may want to use primary cells to address the experimental design.*

Thank you for your comments. We have performed functional experiments to respond your comments using colorectal cancer cell lines, Caco2, HCT116, LoVo, and RKO instead of clinical samples. In these experiments, we assessed effect of mir-143-3p transfection on proliferation of these cells together with transfection reagent alone and transfection with control microRNA. Although there are several reports regarding effect of mir-143-3p on proliferation of colorectal cancer cells, the methods and results were different among the reports. Our results were most similar with that reported by Luo et al, which was added in References No. 32).

We added the sentences below in Abstract, Material and methods, Results, and Discussion.

**Abstract, Line 46-47**

MiR-143-3p transfection significantly inhibited colorectal cancer cell proliferation compared to control microRNA transfection.

**Introduction, Line 83-84**

We also assessed the effect of the candidate miRNA on proliferation of colorectal cancer cells.

**Material and methods, Line 165-189**

**Cell culture**

Human CRC cell lines, Caco2, HCT116, LoVo, and RKO were kind gifts from Dr. Hirofumi Yamamoto (Osaka University Graduate School of Medicine, Osaka, Japan ). These cells were authenticated by ATCC service. These cells except for RKO were cultured by Roswell Park Memorial Institute 1640 medium with 10% fetal bovine serum (FBS). RKO was cultured by Dulbecco’s modified Eagle’s medium with FBS. Culture media and FBS was purchased from Nacalai Tesque (Kyoto, Japan) and Atlas Biologicals (CO, USA) , respectively. Cell experiments were maintained at 37℃ in a humidified atmosphere of 5% CO_2_.

**Cell transfection**

Cells were seeded in 96-well flat-bottom plate at a density of 5000 cells/well for Caco-2, 500 cells/well for HCT116, 1000 cells/well for LoVo, and 500 cells/well for RKO, respectively. 24 hours after seeding, cells were treated with Lipofectamine 2000 and Opti-MEM containing 5 pmol of miRNA Mimic Negative Control (Control treatment), 5 pmol of hsa-miR-143-3p (miR-143 treatment), and no miRNA (Mock treatment) according to the manufacturer’s instructions. Transfection reagents including miRNAs were purchased from Thermo Fisher Scientific.

**Cell proliferation assay**

In vitro growth was evaluated using alamarBlue Cell Viability Reagent at one, two, three, and four days after transfection. The ration of the fluorescence intensity at 590 nm by treatment groups compared to Mock treatment was calculated. Effect of miR-143-3p on cell growth was evaluated by comparison of the ratio between miR-143 treatment and Control treatment using Student’s t-test.

**Results, Line 267-283**

**Effect of miR-143-3p on the proliferation of colorectal cancer cells**

　We assessed the effect of miR-143-3p on the proliferation of CRC cell lines (Caco2, HCT116, LoVo, and RKO). Except for Caco2, miR-143 treatment showed significant growth inhibition on these cell lines compared to Control treatment at four days after transfection (p < 0.01, Fig. 4A, B, C, D). MiR-143 treatment also showed significant inhibition of HCT116 and LoVo compared to Control treatment at three days after transfection (p < 0.01, Fig. 4B and 4C). In Caco2, effect of miR-143-3p on proliferation was not significant at four days after transfection (Fig. 4A, P = 0.06).

Fig. 4. The effect of miR-143-3p on the proliferation of colorectal cancer cells.

The effect of miR-143-3p on the cell growth was evaluated at one, two, three, and four days after transfection. There was no significant effect on proliferation of Caco2 (A). MiR-143-3p showed significant effect on proliferation of HCT116 (B) and LoVo (C) at three and four days after transfection (p < 0.01). MiR-143-3p showed significant effect on proliferation of RKO (D) at four days after transfection (p < 0.01). Results are expressed as mean of the ratio of cell proliferation compared to transfection without miRNA ± standard deviation. * p <0.01 compared to Control treatment.

**Discussion, Line 296-299**

Our data showed that transfection of miR-143-3p inhibited proliferation of CRC cell lines, although the extent of growth inhibition differed among these cell lines. Our results are similar to the previous reports, especially by Luo et al [26,30,32].

**Discussion, Line 306-308**

However, transfection of miR-143-3p should be more effective on cell proliferation than plasma miR-143-3p.

**Discussion, Line 320-321**

First, biological effect of miR-143-3p have not been evaluated by using colonic samples derived from FAP patients or *APC*-deficient mice, although we have shown inhibitory effect of miR-143-3p on proliferation by using CRC cell lines.

***Reviewer #2:***

*While the authors have addressed many of the reviewer comments, the rationale for this study is still lacking.*

Thank you for your comment. Our rationale is described in Introduction as below.

“Circulating miRNAs are recognized as diagnostic or prognostic biomarkers of many diseases [3]; however, their usefulness in the diagnosis of gastrointestinal hereditary diseases has not been reported [4–6]. Genetic variants in patients with hereditary disease may induce consistent and specific miRNA profile differences in their blood compared to healthy donors.”

“However, we hypothesized that plasma miRNA profile of FAP patients, which bears *APC* variants in all cells, may differ from that of patients with sporadic CRC and healthy donors.”

However, these sentences could not introduce the readers what we have done in this study as your comments. We revised Abstract and added the sentences below in Introduction.

**Abstract**

(Previous version) **Line 28-31**

Circulating microRNAs (miRNAs) are considered promising biomarkers for diagnosis, prognosis, and treatment efficacy of malignancies. We assessed circulating miRNAs of patients with familial adenomatous polyposis (FAP) as a hereditary disease and prototype of colorectal cancer progression to evaluate as possible biomarkers of hereditary gastrointestinal diseases.

(Revised version) **Line 30-33**

Circulating microRNAs (miRNAs) are considered promising biomarkers for diagnosis, prognosis, and treatment efficacy of diseases. However, usefulness of circulating miRNAs as biomarkers for hereditary gastrointestinal diseases have not been confirmed yet. We explored circulating miRNAs specific for patients with familial adenomatous polyposis (FAP) as a representative hereditary gastrointestinal disease.

**Introduction**

(Revised version) **Line 81-84**

In this study, we explored the candidate plasma mir-RNAs specific for FAP patients by Next-generation sequencing (NGS) at first and validated the candidate miRNA by further experiments using clinical samples. We also assessed the effect of the candidate miRNA on proliferation of colorectal cancer cells.

*Profiling for FAP patients would be done when? Would this happen after they have polyps? If that's the case, then based on age and other parameters, APC tests might be done. Lynch syndrome and FAP have different phenotypes, both of which are different than non-genetic CRC. This has to be clarified well.*

Thank you for comments regarding the diagnosis of FAP. We diagnosed FAP by clinical features, not by genetic test, because cost of genetic test is not supported by insurance in Japan. Then, the patients are charged several thousand dollars for genetic test. Then, the patients usually do not request for genetic test. Endoscopic findings of stomach (gastric polyp) and duodenum (duodenal polyp) are helpful for distinguish between FAP and Lynch. Different from FAP, genetic test for Lynch is supported by insurance in Japan.

In this study, 16 of 18 patients who had gastric polyps and/or duodenal polyps could be diagnosed as FAP, not Lynch, without genetic test.

We have revised the sentences as below.

**Material and methods**

(Previous version) **Line 83-86**

Ethylenediaminetetraacetic acid containing plasma samples were collected from 18 patients clinically diagnosed endoscopically with FAP. The patients included those with and without a family history of FAP and were treated at our hospital between 2015 and 2018.

(Re-revised version) **Line 90-95**

Ethylenediaminetetraacetic acid containing plasma samples were collected from 18 patients who were diagnosed as FAP by clinical features including family history and endoscopic findings in not only colon and rectum but also stomach and duodenum. Genetic tests for diagnosis of FAP have not been performed in this study, because these tests were not covered by insurance in Japan. These patients were treated at our hospital between 2015 and 2018.
